# Bioactive Compounds and Health-Promoting Properties of Pear (*Pyrus communis* L.) Fruits

**DOI:** 10.3390/molecules25194444

**Published:** 2020-09-27

**Authors:** Joanna Kolniak-Ostek, Dagmara Kłopotowska, Krzysztof P. Rutkowski, Anna Skorupińska, Dorota E. Kruczyńska

**Affiliations:** 1 Department of Fruit, Vegetable and Plant Nutraceutical Technology, Wroclaw University of Environmental and Life Sciences, Chelmonskiego 37 Street, 51-630 Wroclaw, Poland; 2 Laboratory of Experimental Anticancer Therapy, Institute of Immunology and Experimental Therapy, Polish Academy of Sciences, Weigla 12 Street, 53-114 Wroclaw, Poland; dagmara.klopotowska@hirszfeld.pl; 3 Research Institute of Horticulture, Konstytucji 3 Maja 1/3 Street, 96-100 Skierniewice, Poland; krzysztof.rutkowski@inhort.pl (K.P.R.); anna.skorupinska@inhort.pl (A.S.); dorota.kruczynska@inhort.pl (D.E.K.)

**Keywords:** UPLC-MS, pear, bioactive compounds, health-promoting properties, suitability for processing

## Abstract

Consuming food that is rich in antioxidants reduces the risk of developing chronic diseases and oxidative stress. Fruits and vegetables are an excellent source of substances with antioxidant and pro-health properties. Such raw materials, characterized by a high content of polyphenolic compounds and antioxidant capacity, include pear fruits. In this study, the concentrations of bioactive compounds, as well as the antioxidant, anti-inflammatory, and antiproliferative activity in fruits of five selected pear cultivars were determined and compared. LC–MS and UPLC–PDA methods were used to determine the polyphenolic, carotenoid, chlorophyll, and triterpenoid profiles and content, and the antioxidant activity was analyzed using DPPH and ferric-reducing ability of plasma (FRAP) tests. The anti-inflammatory activity was determined against COX-1 and COX-2 enzymes. The cytotoxic activity of the test compounds was assessed against six tumor cell lines. The results showed that the major group of phenolic compounds in all cultivars was phenolic acids. In the group of chromoplastic pigments, chlorophyllide a and 9-*cis*-β-carotene were the major compounds, while in the triterpene group, ursolic acid was dominant. The antioxidant potential correlated with the content of polyphenols and carotenoids, and was the strongest for the ‘Radana’ cultivar. The highest antiproliferative activity in all varieties was established for bladder cancer.

## 1. Introduction

In recent years, more and more attention has been paid to the impact of food on the human body. The development of chronic diseases, autoimmune and neurodegenerative diseases, metabolic diseases, and cancer is positively correlated with oxidative stress [1]. Oxidative stress, that is, the imbalance of antioxidants and prooxidants in favor of prooxidants, is caused by high levels of reactive oxygen species (ROS). In free radical processes, reactive oxygen species react with cellular components, causing modifications and damaging them. Epidemiological studies provide evidence that consuming food that is rich in antioxidants reduces the risk of developing chronic diseases and oxidative stress [2].

Fruits and vegetables are an excellent source of substances with antioxidant and pro-health properties. Such substances include polyphenols, carotenoids, and triterpenoids. Phenolic compounds possess strong antioxidant, anti-inflammatory, antiviral, and anticarcinogenic properties [3,4]. Their presence is associated with a reduced risk of cardiovascular diseases, cancer, diabetes, Alzheimer’s disease, and atopic dermatitis [4,5,6]. Pentacyclic triterpenes are widely distributed in plants and are the subject of numerous phytochemical and pharmacological studies. Triterpene compounds possess antioxidant, anti-inflammatory, and anticancer properties, among others [7,8]. Plants with a high content of pentacyclic triterpenes are often used in herbal medicine due to their valuable therapeutic properties. Carotenoids have valuable biological properties, among which, the most documented is the activity of provitamin A. Carotenoids are classified as both prevention and intervention antioxidants. In the human body, they perform many important functions since they prevent atherosclerosis, maintain the health of the eyes and skin, and reduce the risk of cancer [9,10].

Proposals for alternative raw materials with a desirable effect on the body seem to be justified due to the growing interest of consumers and processors for new materials with health-promoting properties. Such raw materials, characterized by a high content of polyphenolic compounds and antioxidant capacity, include pear fruits (*Pyrus communis* L.), which have been used in herbal medicine for years.

Despite the growing crop of pears, these raw materials have not been described to date in the literature in terms of detailed bioactive properties. There is also limited information about the content of compounds such as carotenoids, chlorophylls, and triterpenoids. The variety of polyphenolic compounds and the high antioxidant capacity of pears mean that these fruits can have a significant impact on the health of the human body. This has become a premise for research on the impact of variations of the content of bioactive compounds and their antioxidant, anti-inflammatory, and antiproliferative activities.

## 2. Results and Discussion

### 2.1. Physicochemical Properties of Pear Fruits

The results of the basic properties of the pear cultivars investigated are presented in Table 1. Fruits of the ‘Nojabrska’ variety were the biggest and ‘Radana’ were the smallest. Paprstein [11] reported that the typical weight of ‘Radana’ fruits is ≈164 g. The total soluble solids content varied from 9.35 (‘Hortensia’) to about 12.5 for ‘Conference’ and ‘Nojabrska.’ The titratable acidity varied from 0.15 (‘Conference’) to 0.47 (‘Radana’). The soluble solids content and titratable acidity varied from season to season and changed during storage [12,13,14]. The flesh firmness, starch index, and ethylene and carbon dioxide production indicated that the fruits were unripe but were suitable for storage [13,14]. Kappel at al. [15] suggested that the firmness and soluble solids content of pears that are ideal for consumption should be in the range of 18 to 22 N and above 14%, respectively. Konopacka et al. [12] suggested that each cultivar needs an individual strategy during storage and ripening to obtain the best sensory attributes. 

### 2.2. Quantification of the Phenolic Compounds in the Pear Fruits

**Figure 1:**
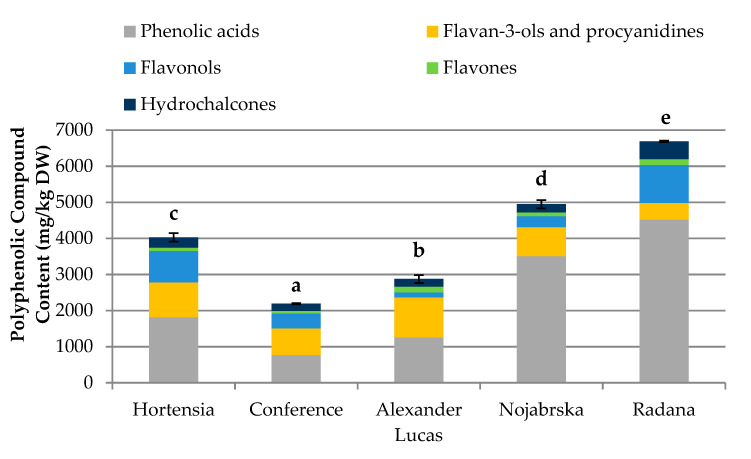
Content of polyphenolic compounds (mg/kg dry weight (DW)) in the pear cultivars. Varieties with different letters were significantly different at *p* = 0.05 according to Duncan’s test. The data are expressed as mean ± SD (*n* = 9).

Figure 1 and Table S1 show the data after the UPLC-MS and phloroglucinolysis analysis. 

The concentration of phenolic compounds in the selected pear cultivars ranged from 2188.93 mg/kg dry weight (DW) (‘Conference’) to 6687.71 mg/kg DW (‘Radana’). ‘Nojabrska’ and ‘Radana’ cultivars had greater phenolic contents than the average of 4438.32 mg/kg DW (Table S2). The highest concentrations of phenolic acids (4521.15 mg/kg DW), flavonols (1056.20 mg/kg DW), and hydrochalcones (493.56 mg/kg DW) were found in the ‘Radana’ pears, while the ‘Nojabrska’ cultivar was characterized by the highest content of flavan-3-ols (1108.74 mg/kg DW) and flavones (160.70 mg/kg DW).

The content of phenolic compounds in fruits is affected by the environmental growth, variety, and maturity stage. Additionally, the phenolic compounds are not equally distributed within the fruit [16]. The peel is rich in anthocyanins, flavonols, catechins, and procyanidins, while hydroxycinnamic acids are mainly present in the core.

Considerably variable amounts of phenolics in different pear cultivars were reported previously [16,17]. In research on the ‘Conference’ variety, Cebulak et al. [18] found significantly higher levels of phenolic compounds (3190.0 mg/100 g DW), while results obtained by Liaudanskas et al. [19] were much lower (710 mg/kg DW). Compared to other stone fruits, the polyphenolic content in pears is similar to that in apples (1654.8–5314.1 mg/kg DW) [20] and quince (2609.50 mg/100 g DW) [21].

**Figure 2:**
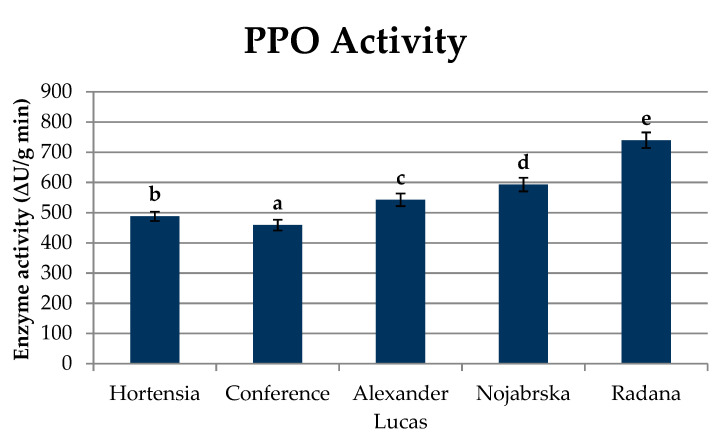
Polyphenol oxidase (PPO) activity (∆U/g min) in pear cultivars. Varieties with different letters were significantly different at *p* = 0.05 according to Duncan’s test. The data are expressed as mean ± SD (*n* = 9).

The concentration of polyphenols in fruits is influenced by the activity of the enzyme polyphenol oxidase (PPO; E.C. 1.14.18.1), which in the presence of oxygen, catalyzes the processes of oxidation of monophenols to o-diphenols and the corresponding o-quinones, and then to brown melanins, as well as the reaction of polymerization and condensation of proteins with polyphenols, which results in an undesirable change in the color of plant tissues [22]. Pear fruits are susceptible to enzymatic oxidation reactions, which can be observed during the shredding process. Studies have shown that PPO activity is significantly different in the tested varieties of pears (Figure 2, Table S1). 

Polyphenol oxidase activity ranged between 458.94 ∆U/g min (‘Conference’) and 739.80 ∆U/g min (‘Radana’) (Figure 2). In comparison to other fruits, the PPO activity in pears was similar to that in apples (270–3120 ∆U/g min) [23] and quince (709.85 to 1284.59 ∆U/g min) [24]. Pear fruits are susceptible to enzymatic oxidation reactions, which manifest rapid browning during grinding. Amiot, Tacchini, Aubert, and Nicoas [25] report that high PPO activity is conditioned by the presence of chlorogenic acid and compounds from the flavan-3-ol group. In contrast, flavonols and hydrochalcones are only slightly affected by this reaction. The obtained results confirm that monomeric catechins were the main substrates of the enzymatic browning reaction. This was indicated by the high correlation coefficients between the content of individual polyphenolic compounds and polyphenol oxidase activity (R^2^ = 0.79 for monomeric catechins and R^2^ = 0.80 for polymeric procyanidins) (Figure S1). In the case of caffeic acid and its derivatives, the correlation coefficient was positive and amounted to R^2^ = 0.79. The lowest correlation coefficients were calculated for isorhamnetin and its derivatives (R^2^ = 0.05) and for quercetin and its derivatives (R^2^ = 0.10). In the case of hydrochalcones, the correlation coefficient was found to be R^2^ = 0.69 (Figure S1).

The determination of PPO activity is important for determining the suitability of fruit for processing. The industry is looking for varieties with a low PPO activity due to the use of a technological treatment where tissue is torn and the polyphenolic compounds released from the cell are oxidized in the presence of oxygen. In the case of fruit with a high activity of oxidizing enzymes and a significant content of reaction substrates, it is necessary to select and use substances that prevent these changes. 

### 2.3. Quantification of the Triterpenoids in Pear Fruits

**Figure 3:**
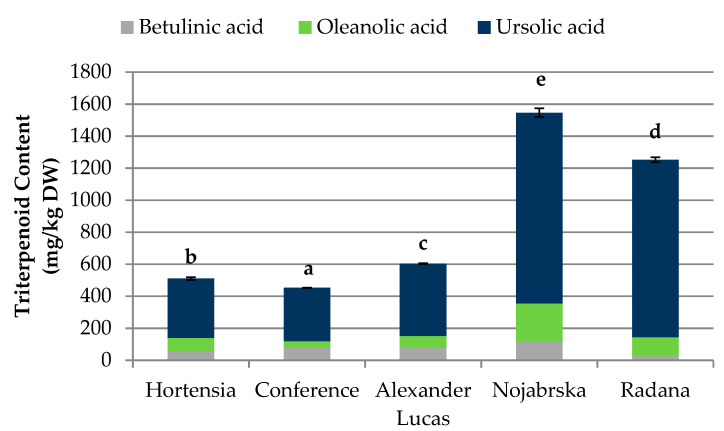
Content of triterpenoids (mg/kg DW) in pear cultivars. Varieties with different letters were significantly different at *p* = 0.05 according to Duncan’s test. The data are expressed as mean ± SD (*n* = 9).

Various levels of betulinic, oleanolic, and ursolic acids were found in the selected pear cultivars (Figure 3, Table S2). 

The highest content of triterpenes was found in the ‘Nojabrska’ cultivar (1546.17 mg/kg DW), and the lowest was found in the ‘Conference’ pears (452.71 mg/kg DW). The most abundant triterpene compound in all examined fruits was ursolic acid. This was in agreement with the results obtained by Sun et al. [26]. In their study on triterpenoids in thinned young fruits of ten pear varieties, among 16 identifies triterpenoids, ursolic acid was predominant. The ‘Nojabrska’ cultivar was characterized by the highest concentration of all the investigated triterpenes. The content of betulinic, oleanolic, and ursolic acids was 116.12 mg/kg DW, 239.34 mg/kg DW, and 1190.71 mg/kg DW, respectively. The lowest concentration of betulinic acid (25.90 mg/kg DW) was found in the ‘Radana’ cultivar, while ‘Conference’ pears were characterized by the lowest content of oleanolic (41.16 mg/kg DW) and ursolic (333.92 mg/kg DW) acids (Figure 3, Table S2).

Compared to earlier research, the concentration of triterpenes in the examined fruits was lower than in the pear peel (3460.5 mg/kg DW) but higher than in the pear pulp (201.4 mg/kg DW) [27]. Furthermore, other researchers found significant differences between the triterpenoid concentrations in different pear anatomical parts [16,28]. Triterpenes are generally found in the cortex, peel, and waxes of leaves, fruits, and flowers, acting as a protector against insect and microbial attack. They are widespread in the world of plants and are the subject of numerous phytochemical and pharmacological studies. Plants with high levels of pentacyclic triterpenes are often used in phytotherapy due to their valuable therapeutic properties. Numerous studies have shown a wide range of pro-health effects of triterpenes, such as antiviral, antitumor, anticancer, cytoprotective, and anti-inflammatory activities [20,21,22,23,24,25,26,27,28,29,30,31].

### 2.4. Quantification of the Carotenoids and Chlorophylls in Pear Fruits

**Figure 4:**
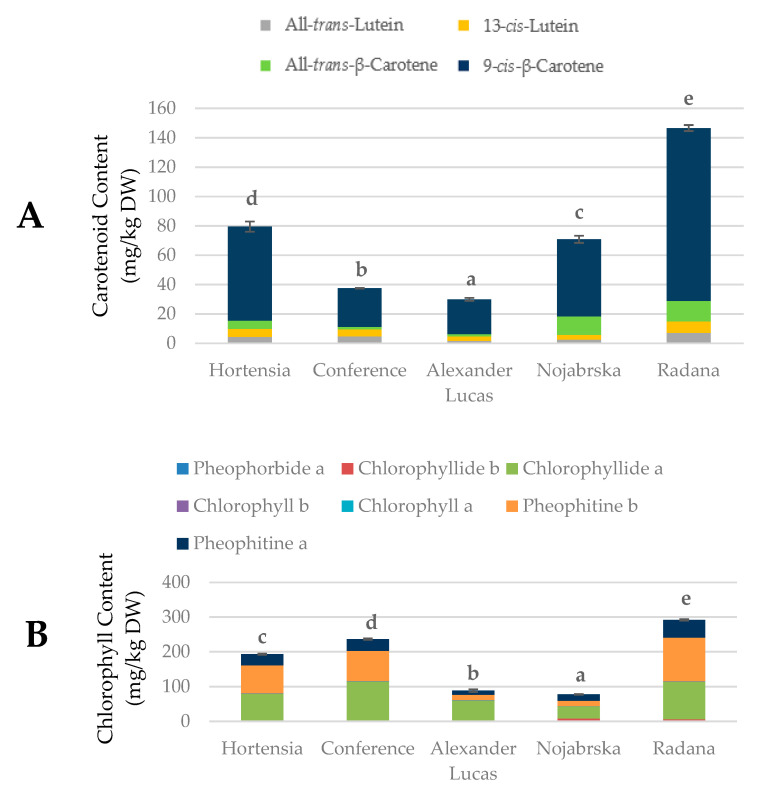
Contents of the (**A**) carotenoids and (**B**) chlorophylls (mg/kg DW) in pear cultivars. Varieties with different letters were significantly different at *p* = 0.05 according to Duncan’s test. The data are expressed as mean ± SD (*n* = 9).

Figure 4 and Table S2 show the data from the UPLC-MS analysis of the chlorophylls and carotenoids in selected pear cultivars. 

The concentrations of chlorophylls ranged from 77.43 mg/kg DW (‘Nojabrska’) to 291.74 mg/kg DW (‘Radana’). The concentrations of carotenoids ranged between 29.91 mg/kg DW (‘Alexander Lucas’) and 146.61 mg/kg DW (‘Radana’). The highest sum of chromoplastic pigments (438.35 mg/kg DW) was found in ‘Radana’ pears (these fruits were characterized by the highest percentage of blush; Table 1), while the lowest (118.47 mg/kg DW) was found in ‘Alexander Lucas.’ The predominant chlorophyll compound in the investigated fruits was chlorophyllide a (mean of 78.08 mg/kg DW), and its highest concentration was found in the ‘Conference’ cultivar. In the group of carotenoids, the predominant compound was 9-cis-β-carotene (mean of 56.86 mg/kg DW). The highest concentration of this compound (117.72 mg/kg DW) was found in the ‘Radana’ pears (Figure 4 and Table S2).

Compared to other fruits, the concentrations of chlorophylls in the selected pear cultivars was similar to that in apples (means of 7.1 g/kg DW in the flesh and 108.48 in the peel) [32]. The concentrations of carotenoids were similar to that in apple flesh (means of 45.22–71.58 mg/kg DW) [30] and lower than in watermelon (mean of 185.00 mg/kg DW) [33].

Variations in the color and appearance of the fruit skin depended on the concentrations of pigments, such as chlorophylls and carotenoids. According to Charoenchongsuk et al. [34], the carotenoid and chlorophyll contents depend on the cultivar and maturation stage. During fruit ripening, chlorophylls are degraded to colorless catabolites, and the carotenoids become perceptible.

### 2.5. Antioxidant Capacity of Pear Fruits

**Figure 5:**
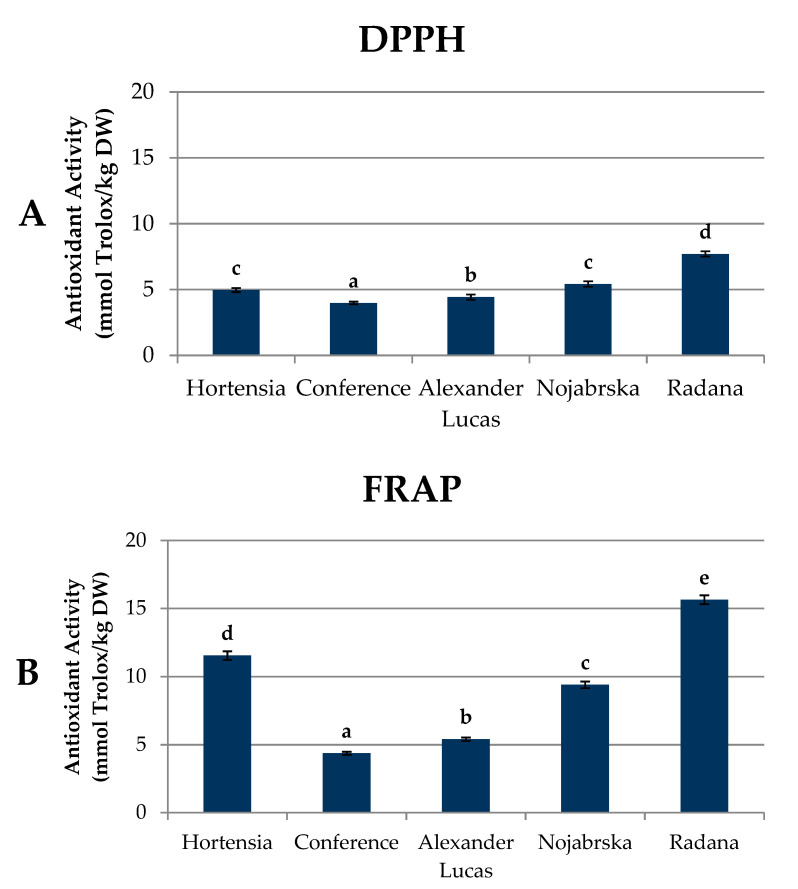
Antioxidant activity of pear fruits (mmol Trolox/kg DW) measured as (**A**) the free radical scavenging activity (DPPH method) and (**B**) ferric reducing capacity using the ferric-reducing ability of plasma (FRAP) method. Varieties with different letters were significantly different at *p* = 0.05 according to Duncan’s test. The data are expressed as mean ± SD (*n* = 9). FRAP: ferric-reducing ability of plasma.

The antioxidant activity was measured as the free radical scavenging activity (DPPH method) and ferric reducing capacity using the ferric-reducing ability of plasma (FRAP) method (Figure 5, Table S3). In this study, the results of the DPPH and FRAP methods were expressed in the same unit, i.e., millimoles of Trolox equivalent (TE) per kg of pear dry weight.

The highest levels of DPPH and FRAP antioxidant activities (7.70 mmol Trolox/kg DW and 15.64 mmol Trolox/kg DW, respectively) were found in the ‘Radana’ fruits, while the ‘Conference’ cultivar was characterized by the lowest results: 3.98 and 4.37 mmol Trolox/kg DW, respectively. The obtained results were lower than those determined by Liaudanskas et al. [19]. In their study on ‘Conference’ pears, the DPPH antioxidant capacity reached over 40 µmol TE/g DW, while the FRAP activity was approximately 30 µmol TE/g DW.

The antioxidant capacity of plant materials is affected by the type and number of bioactive compounds they contain. For biologically active substances found in fruits and vegetables, among others, polyphenol compounds can be included. The strong antioxidant properties of these compounds are connected to the number of hydroxyl groups present in the molecule. Depending on the location and amount of hydroxy, methoxy, and the rest of the glycosides, the biological activity of polyphenolic compounds and their physical and chemical properties differ [35]. Our results suggest that the antioxidant capacity was related to the presence of phenolic compounds (R^2^ = 0.98 for both DPPH and FRAP) (Figures S2 and S3). In the case of the DPPH test, its value was mostly influenced by the catechin and procyanidin polymers (R^2^ = 0.75 and 0.76, respectively). Quercetin and apigenin derivatives (R^2^ = 0.71 and 0.62, respectively) and arbutin (R^2^ = 0.85) had smaller impacts on the value of the DPPH capacity. The lowest degree of correlation was observed between DPPH and the content of sinapic acid and isorhamnetin derivatives (R^2^ = 0.17 and 0.21, respectively) (Figure S2). For the FRAP capacity, the highest degree of correlation was obtained for the quercetin and arbutin derivatives (R^2^ = 0.83 and 0.76, respectively). Slightly lower values were observed for the FRAP and catechin monomers (R^2^ = 0.67). The lowest degree of correlation was obtained for ferulic and sinapic acids (R^2^ = 0.08 in both cases) (Figure S3).

The relation between polyphenolic compounds and the antioxidant capacity in foods has been repeatedly addressed in the literature, but different conclusions were reached on this issue. In some experiments, no correlation in plant extracts was found, while in others, a strong relationship between polyphenols and capacities was found [19,36,37].

Eberhardt, Lee, and Liu [38] reported on the strong antioxidant activity of the polymer procyanidins. Kolniak-Ostek, Oszmianński, and Wojdyło [39] reported that the polyphenolic compounds from the group of flavan-3-ols have the greatest impact on the antioxidant capacity. 

Another group of compounds with proven pro-health activity are carotenoids. Due to the presence of several double bonds in the molecule, carotenoids readily react with electrophiles. Carotenoids are classified as both prevention and intervention antioxidants. The antioxidant capacity of carotenoids increases with the increased extension and maximum overlap of the conjugated double bond molecular orbitals [40]. The obtained results suggest a strong influence of carotenoids on the antioxidant capacity (R^2^ = 0.93 for the DPPH and FRAP tests) (Figures S4 and S5). For the DPPH capacity, the highest correlation was found for 13-*cis*-lutein and 9-*cis*-β-carotene (R^2^ = 0.95 and 0.92, respectively). For the FRAP test, the highest correlation coefficients were found for all-*trans*-lutein and 9-*cis*-β-carotene (R^2^ = 0.88 and 0.94, respectively) (Figures S4 and S5). At the same time, no correlation was found between chlorophylls and the antioxidant capacity (Figures S6–S8).

Despite the reports on the antioxidant properties of triterpenes, which can be found in the literature [41,42], the obtained results did not show a correlation between the content of triterpene compounds and the antioxidant capacity (Figure S8).

### 2.6. Anti-Inflammatory Activity of Pear Fruits

**Figure 6:**
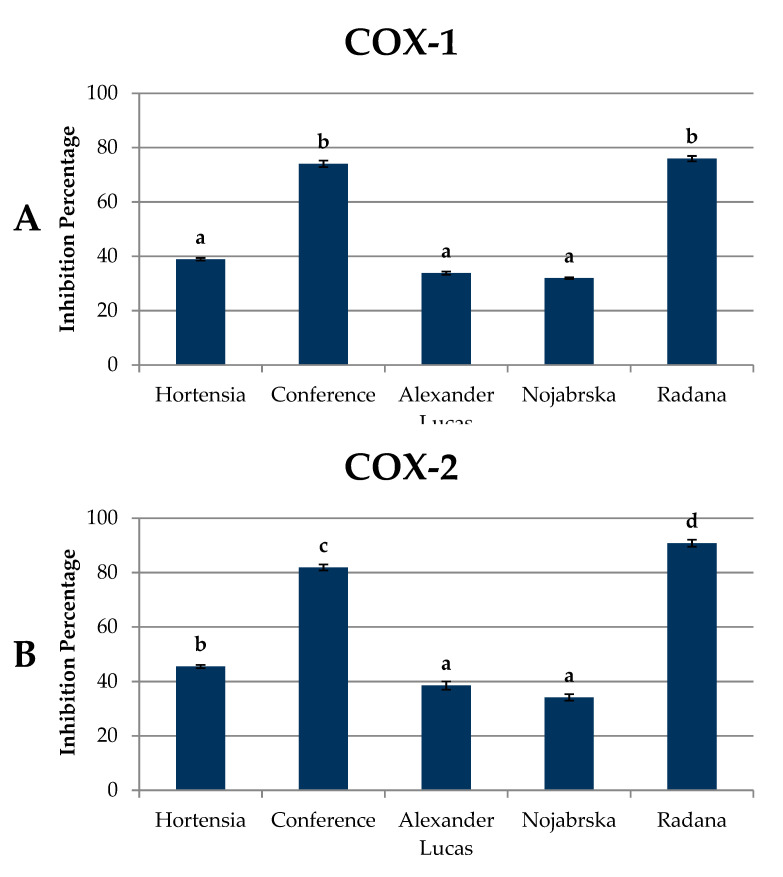
Anti-inflammatory activity of pear fruits (% of inhibition) against (**A**) cyclooxygenase-1 (COX-1) and (**B**) cyclooxygenase-2 (COX-2) enzymes. Varieties with different letters were significantly different at *p* = 0.05 according to Duncan’s test. The data are expressed as mean ± SD (*n* = 9).

Figure 6 and Table S4 show the results of the anti-inflammatory activity (against cyclooxygenase-1 (COX-1) and cyclooxygenase-2 (COX-2)) of pear extracts. 

The mean capacity of the compounds contained in pear extracts to inhibit COX-1 was 50.53%. The mean capacity of the compounds to inhibit the COX-2 enzyme was higher at 58.15%. The highest level of anti-inflammatory activity against COX-1 and COX 2 enzymes was found in the ‘Radana’ and ‘Conference’ polyphenolic extracts. For the COX-2 enzyme, pear extracts were more effective, being characterized by an approximately 15% higher anti-inflammatory activity than for COX-1. Cyclooxygenase-1 and 2 are enzymes that differ in biological activity but exhibit similar catalytic properties. COX-1 is a constitutive cyclooxygenase, which is responsible for maintaining the normal function of internal organs by regulating the synthesis of prostanoids, whereas the induced isoform COX-2 increases its expression under the influence of stress factors and inflammation. This is why compounds that have the ability to inhibit COX-2 are considered to be antitumorigenic [43]. According to Li et al. [11], pear extracts showed anti-inflammatory properties when applied to mouse models of hind paw edema and ear edema. The anti-inflammatory activity of the compounds depends on their molecular structure. In their study, Seeram, Momin, Nair, and Bourquin [44] found that the lower the number of sugar residues attached to the aglycone was, the higher the anti-inflammatory activity was. They noted the highest anti-inflammatory activity against COX-1 and COX 2 (38.7% and 46.8%, respectively) for cyanidin, followed by cyanidin-rutinoside and cyanidin-glucosylrutinoside. 

### 2.7. Antiproliferative Activity of Pear Fruits

**Figure 7:**
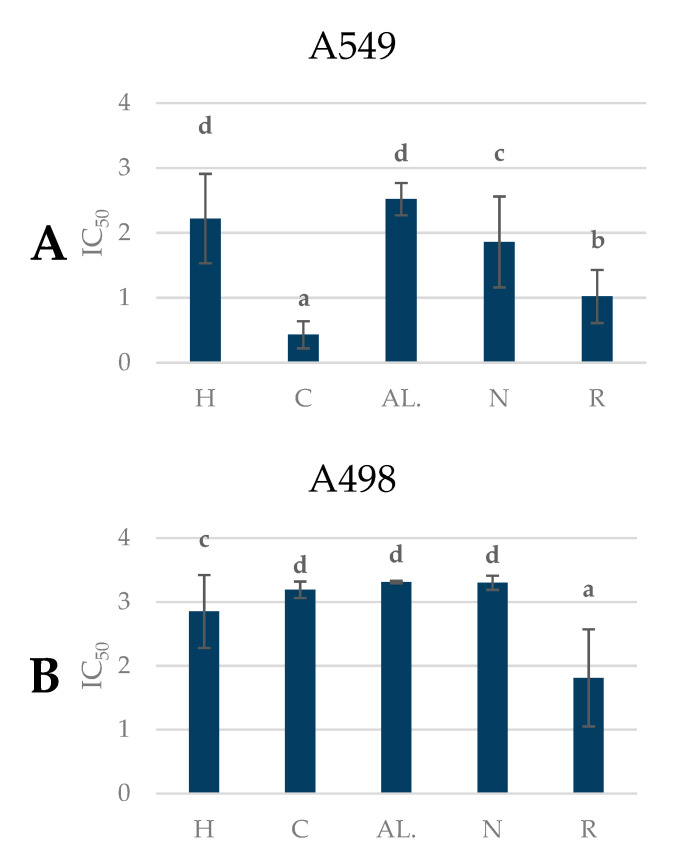

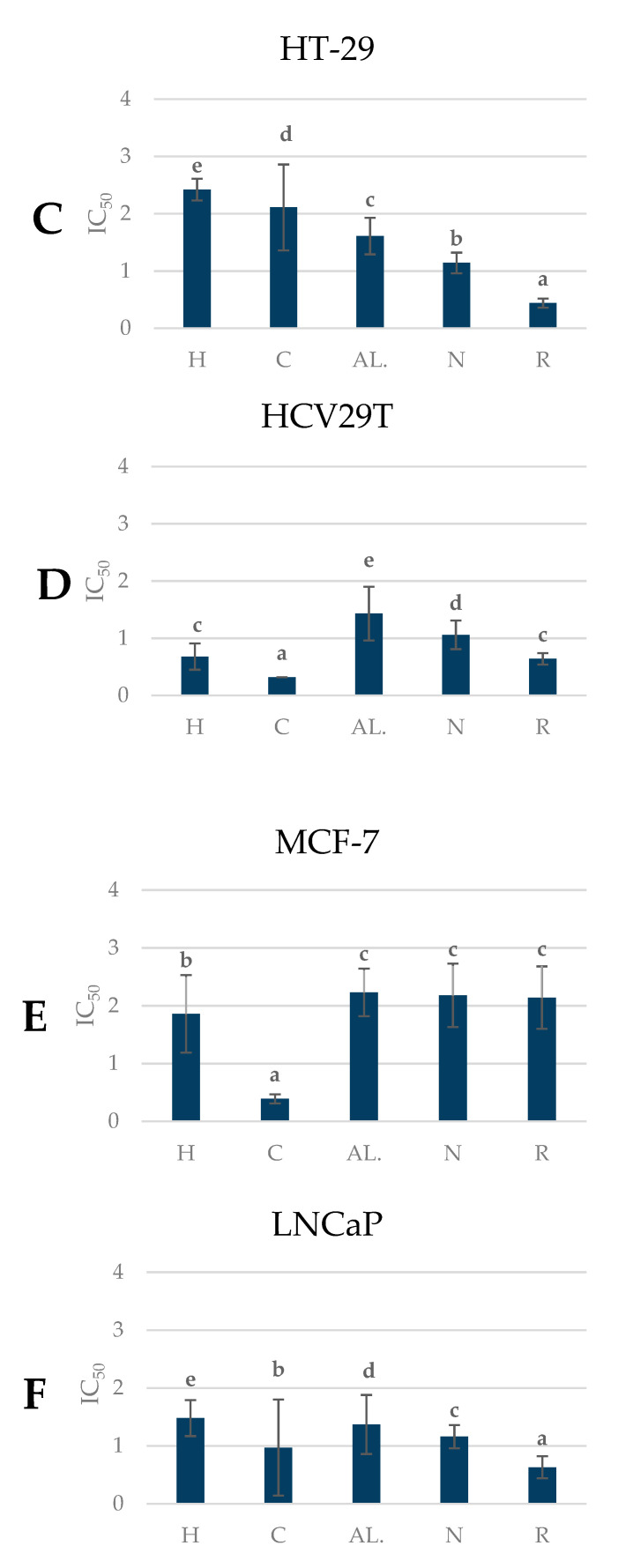
Antiproliferative properties of pear cultivars (IC_50_). Varieties with different letters were significantly different at *p* = 0.05 according to Duncan’s test. The data are expressed as mean ± SD (*n* = 9). H—Hortensia, C—‘Conference’, AL.—‘Alexander Lucas’, N—‘Nojabrska’, R—‘Radana’. (**A**) A549 – human lung cancer; (**B**) A498 – human kidney cancer; (**C**) HT-29’colon adenocarcinoma; (**D**) HCV-29T’bladder cancer; (**E**) MCF-7’human mammary gland cancer; (**F**) LNCaP’prostate cancer.

The antitumor activity of polyphenolic preparations obtained from the pear fruit against cancer cells is presented in Figure 7 and Table S5.

The results of the experiments are presented in terms of IC_50_, which is the concentration causing inhibition of the proliferation of 50% of the cancer cell population. For the analysis of the biological activity of pear fruit, six cancer cell lines of various etiologies were selected: A-498 (human kidney cancer), A-549 (human lung cancer), HCV-29T (bladder cancer), HT-29 (colon adenocarcinoma), LNCaP (prostate cancer), and MCF-7 (human mammary gland cancer).

The tested extracts were characterized by different antiproliferative activities against cancer cells. The highest activity against A498, HT29, and LNCaP cells was demonstrated by the polyphenolic compound extracts from the pear cultivar ‘Radana,’ while the highest activity against cell lines A549, HCV29T, and MCF-7 was shown by the extracts from the ‘Conference’ variety (Figure 7).

The cultivars ‘Radana’ and ‘Conference’ had the highest antiproliferative activity against cancer cells compared to the other cultivars (the lowest IC_50_). The highest antiproliferative activity in all the varieties was established for bladder cancer, with a mean of 0.83 mg/mL.

The obtained results indicate the proliferative effect of the tested pear fruit extracts. Studies have shown that the antiproliferative effects of tumor preparations were dependent on the pear variety. Differences in the inhibition of tumor cell proliferation for different pear cultivars could be related to the concentration and type of polyphenolic compounds of the tested preparations. 

Previously, Živković et al. [45] observed the cytotoxic activity of pear and apple extracts against human epithelial carcinoma cell line (HeLa), human colon carcinoma (LS174), and human fetal lung fibroblast (MRC-5) cells. Furthermore, El-Hawary et al. [46] showed that volatile constituents from Pyrus communis have good cytotoxic and antimicrobial activities. Recent studies have shown that it is the polyphenols contained in the fruit, primarily quercetin, catechins, and chlorogenic acid, that have strong antitumor properties. Pear fruit is rich in polyphenolic compounds, and thus it is a good source of compounds with antiproliferative properties. Increased consumption of fruit and vegetables is recommended as a key component in a healthy diet for the prevention of certain types of cancer [47].

## 3. Materials and Methods 

### 3.1. Reagents and Standards

Acetonitrile, formic acid, methanol, DPPH (1,1-diphenyl-2 picrylhydrazyl radical), Trolox (6-hydroxy-2,5,7,8-tetramethylchroman-2-carboxylic acid), TPTZ (2,4,6-tri(2-pyridyl)-s-triazine), acetic acid, phloroglucinol, arbutin, caffeic acid, betulinic, oleanolic and ursolic acid, fructose, glucose, sorbitol and sucrose, and malic, oxalic, citric, shikimic, succinic, and fumaric acids were purchased from Sigma-Aldrich (Steinheim, Germany). Quercetin 3-*O*-glucoside, kaempferol 3-*O*-glucoside, cyanidin 3-*O*-glucoside, isorhamnetin 3-*O*-glucoside, apigenin 7-*O*-glucoside, chlorogenic, quinic, *p*-coumaric and ferulic acids, (+)-catechin, and procyanidin B2 were purchased from Extrasynthese (Lyon, France). Sodium hydroxide (CAS 1310-73-2), iodine (CAS 7553-56-2), and potassium iodide (CAS 7681-11-0) were purchased from CHEMPUR (Piekary Slaskie, Poland).

### 3.2. Plant Material 

Pear fruits (*Pyrus communis* L.) of the following cultivars: ‘Hortensia’, ‘Konferencja’ (‘Conference’), ‘Lukasówka’ (‘Alexander Lucas’), ‘Nojabrska,’ and ‘Radana’ were used in this study. ‘Hortensia’ (on the market since 1996) late autumn German cultivars (ripen a few days before ‘Conference’), crossing of ‘Nordhäuser Winterforelle’ × ‘Clapps Favourite’; ‘Konferencja’ (‘Conference’) late autumn cultivars found as an open-pollinated seedling from a ‘Leon Leclerc de Laval’ in 1884; ‘Lukasówka’ (‘Alexander Lucas’) winter cultivars that originated as a chance seedling in the Department of Loire et Cher, France, in 1866; ‘Nojabrska’ (‘Xenia’) old Moldavian late autumn cultivars, crossing of ‘Triumphe de Vienne’ × ‘Krier’; ‘Radana’ summer Czech cultivars that were registered in 1994, crossing of ‘Louise bonne d’Avranche’ × ‘Clapp’s Favourite.’ Fruits of ‘Hortensia’ and ‘Radana’ are characterized by an attractive red blush, while fruits of the other cultivars are green without the blush. Fruits were collected in 2014 from the Experimental Orchard belonging to the Research Institute of Horticulture in Skierniewice, Poland (51°55′24′′ N, 20°5′58′′ E). The fruits were harvested at the ripening stage recommended for storage (based on the ethylene and carbon dioxide production, firmness, and the starch index; Table 1).

The measurement of the maturity and quality parameters were carried out on samples of 30 fruits (taken from three trees for 10 fruits from each tree). Additionally, for the chemical analyses (for each variety), approximately 3 kg of fruits were collected. For each variety, three independent samples were made and analyzed three times (*n* = 9). 

Measurements of the fruit weight, percentage of blush, total soluble solids (TSS) content, titratable acidity (TA), flesh firmness, and fruit maturity (ethylene and carbon dioxide productions and starch index) were conducted on fresh pears soon after harvest. For the polyphenolic compounds, carotenoids, triterpenoids, antioxidant capacity, and anti-inflammatory activity analyses, the whole fruits were cut and directly frozen in liquid nitrogen and freeze-dried (24 h; Christ Alpha 1–4 LSC, Martin Christ GmbH, Osterode am Harz, Germany). Homogeneous powders were obtained by crushing the dried tissues using a closed laboratory mill to avoid hydration (IKA 11A, Staufen, Germany). The powders were kept in a refrigerator (−80 °C) until the analyses. For the cytotoxic activity analyses, crushed fruits with the addition of an antioxidant (NaHSO_3_, 200 mg·kg^−1^) were pressed on a hydraulic press, after which, the obtained juice was passed through the resin for the adsorption of polyphenolic compounds. After applying the juice to the column with an Amberlite XAD 16 (Rohm & Haas, Philadelphia, PA, USA), the organic acids and sugars were eluted with redistilled water and polyphenolic compounds were eluted with 96% ethyl alcohol. The ethyl alcohol was removed using rotary evaporation in a vacuum evaporator at 40 °C, and the residue was freeze-dried for 24 h. The obtained polyphenolic compound preparation was kept in a refrigerator (−80 °C) until the analyses.

### 3.3. Physicochemical Analyses

The fruit weight was determined by weighing individual fruits on a WPS2100/C/2 laboratory balance (Radwag, Radom, Poland). The measurement results are expressed in grams (g). The TSS content (in juice collected from individual fruits) was measured with an Atago PR-101 digital refractometer (Atago Co. Ltd., Tokyo, Japan) and expressed as °Brix. The TA was measured using a DL 21 automatic titrator (Mettler-Toledo, Schwerzenbach, Swiss) via the standard titration method, 0.1 N NaOH, pH = 8.1 end point, and expressed as a percentage. To measure the production rates of carbon dioxide and ethylene, the pears were individually placed in 1.8 L glass jars equipped with septa. The jars were hermetically closed for two hours and then two samples of 1 mL each were taken (one for ethylene and the second for carbon dioxide). The carbon dioxide was measured using an ADC 225 MK3 gas analyzer (ADC The Analytical Development Co. Ltd, Hoddesdon, UK), while ethylene was measured using an HP 5890 II gas chromatograph (Hewlett Packard, Wilmington, DE, USA) equipped with an alumina-packed glass column. The results are expressed in µL·kg^−1^·h^−1^ (ethylene production rate) and µL·g^−1^·h^−1^ (carbon dioxide production). The percentage of blush was assessed subjectively (bonitation) and expressed as a percentage. The flesh firmness was measured on two opposite sides of each fruit using an EPT-1R pressure tester (Lake City Technical Products, Inc, Kelowna, Canada), equipped with an 8 mm tip. The starch index was determined using the ten-point scale in the standard iodine test (1 = black, 10 = white).

### 3.4. Identification and Quantification of the Polyphenols Using the UPLC-PDA-MS Method

For the extraction and determination of the polyphenols, a protocol described before by Kolniak-Ostek [27] was followed. The column used was a UPLC BEH C18 column (1.7 µm, 2.1 mm × 100 mm; Waters Corp., Milford, MA, USA). The mobile phase consisted of aqueous 0.1% formic acid (A) and 100% acetonitrile (B). All incubations were done in triplicate. The results are expressed as milligrams per gram of DW.

### 3.5. Analysis of the Proanthocyanidins Using the Phloroglucinolysis Method

Direct phloroglucinolysis of the freeze-dried pear varieties was performed as described previously by Kolniak-Ostek [27]. The phloroglucinolysis products were separated on a Cadenza CD C18 (75 mm × 4.6 mm, 3 µm) column (Imtakt, Kyoto, Japan). Solvent A (25 mL of aqueous acetic acid and 975 mL of water) and solvent B (acetonitrile) were used. The compounds for which the reference standards were available were identified in chromatograms according to their retention times and UV-visible spectra. The fluorescence detection was recorded at the excitation wavelength of 278 nm and the emission wavelength of 360 nm. All incubations were done in triplicate. The results are expressed as milligrams per kilogram DW.

### 3.6. Analysis of the Triterpenoids Using the UPLC-PDA-MS Method

For the extraction and determination of the triterpenoids, a protocol described earlier was followed [48]. The separations of individual triterpenoids were carried out using a UPLC BEH C18 column (1.7 mm, 2.1 mm × 150 mm; Waters Corp.) at 20 °C. The elution solvents were 100% methanol (A) and 100% acetonitrile (B) (15:85, *v*/*v*). Ursolic, oleanolic, and betulinic acids were eluted isocratically at a flow rate of 0.1 mL·min^−1^ for 10 min. All incubations were done in triplicate. The results are expressed as milligrams per kilogram DW.

### 3.7. Analysis of the Carotenoids and Chlorophylls Using the UPLC-PDA-MS Method

For the extraction and determination of carotenoids and chlorophylls, a protocol described earlier was followed [48]. The compounds were separated with an ACQUITY UPLC BEH RP C18 column (1.7 µm, 2.1 mm × 100 mm; Waters Corp.) at 32 °C. The elution solvents were ACN:MeOH (7:3, *v*/*v*) (A) and 0.1% formic acid (B). All incubations were done in triplicate. The results are expressed as milligrams per kilogram DW.

### 3.8. Analysis of the Antioxidant Activity

The total antioxidant potential of samples was determined using a FRAP assay from Benzie and Strain [49] as a measure of the antioxidant power. The DPPH radical scavenging activity of samples was determined according to the method of Yen and Chen [50]. A standard curve was prepared using different concentrations of Trolox. All determinations were performed in triplicate using a Shimadzu UV-2401 PC spectrophotometer (Kyoto, Japan). The results were corrected for dilution and are expressed in milioles of Trolox equivalent per 100 grams of DW.

### 3.9. Analysis of the Polyphenol Oxidase Activity

The PPO activity was measured following the method previously described by Gonzalez, de Ancos, and Cano [51]. The PPO activity was determined by measuring the initial rate of increase in absorbance after the reaction at 420 nm. The activity was assayed with a Shimadzu UV-1800 spectrophotometer (Kyoto, Japan). The enzyme activity was determined by measuring the slope of the reaction curve at zero time (initial rate) and after 2 min of reaction time. The enzyme activity unit was defined as the difference in absorbance per minute and gram of tissue. 

### 3.10. Anti-Inflammatory Activity

The anti-inflammatory activity was determined using a method described by Mizgier, Kucharska, Sokół-Łętowska, Koniak-Ostek, Kidoń, and Fecka [52], which consists of spectrophotometric measurements of the inhibition of cyclooxygenase (COX-1, COX-2) at a wavelength of 611 nm. The measurements were recorded on a Shimadzu UV-1800 spectrophotometer (Kyoto, Japan) over 3 min. The inhibition percentage of cyclooxygenase activity was calculated using the following formula:

$$\%\;\mathrm{Inhibition}=\frac{{\Delta A}_{\mathrm{control}}-{\Delta A}_{\mathrm{sample}}}{{\Delta A}_{\mathrm{control}}}\;\times 100\%{,}_{\;}$$

where ΔA_control_ and ΔA_sample_ denote the increases of absorbance 3 min after the substrate addition to the probe without or with the extract tested, respectively.

### 3.11. Antiproliferative Assay In Vitro

Six human cancer cell lines established in vitro using the method described by Wietrzyk, Chodyński, Fitak, Wojdat, Kutner, and Opolski [53] were applied, namely, colon HT-29, breast MCF-7, prostate LNCaP, urinary bladder HCV29T, kidney A498, and lung A549 cell lines.

Extracts of the polyphenolic compounds were obtained from the same amount of each fruit cultivar, although they differed in the composition and concentration of polyphenolic compounds, as shown in Table 1. For the antiproliferative tests, 50 mg DW of the preparation was dissolved in 2.5 mL of culture medium (20 mg/mL). This was the same amount for all pear varieties. 

The antiproliferative activity was determined using a method described by Kłopotowska, Matuszczyk, and Wietrzyk [54], and was defined as the IC_50_. The sulforhodamine B (SRB) assay was used to determine IC_50_ (the half-maximal inhibitory concentration). Briefly, cancer cells were seeded at a density of 10,000 cells per well in 96-well cell culture clusters one day before the assay and maintained at 37 °C in 5% CO_2_. Then, the cells were treated with extracts of polyphenolic compounds in four concentrations ranging from 10 to 0.01 mg/mL for 72 h. After the incubation, the cells were fixed with cold 50% (*w*/*v*) trichloroacetic acid (Avantor Performance Materials, Gliwice, Poland) at 4 °C for 1 h, rinsed with tap water, and stained with 0.4% (*w*/*v*) solution of SRB (Sigma-Aldrich, Darmstadt, Germany) dissolved in 1% (*v*/*v*) acetic acid (Avantor Performance Materials, Gliwice, Poland) for 30 min. The plates with stained cells were rinsed with 1% (*v*/*v*) acetic acid and air-dried at room temperature. The protein-bound dye was extracted from the stained cells with a 10 mM TRIS base (Avantor Performance Materials) solution, and the absorbance at 540 nm was measured using a Biotek Hybrid H4 reader (BioTek Instruments, Winooski, VT, USA). The inhibition of the cell growth in the test sample is expressed relative to the control as a percentage of the untreated control and was calculated using:

$$\left(\frac{\left(\mathrm{absorbance}\;\mathrm{of}\;\mathrm{treated}\;\mathrm{sample}-\mathrm{background}\;\mathrm{absorbance}\right)}{\left(\mathrm{absorbance}\;\mathrm{of}\;\mathrm{untreated}\;\mathrm{control}-\mathrm{background}\;\mathrm{absorbance}\right)}\times \;100\right)-100$$

In each experiment, the samples containing specific concentrations of the preparation were applied in triplicate. The experiments were repeated three times. The IC_50_ values were calculated using Cheburator 0.9.0 software (www.cheburator.nevozhay.com).

### 3.12. Statistical Analysis 

The results are presented as the mean of three technical replications. Statistical analyses were performed with Statistica version 12.5 (StatSoft, Tulsa, OK, USA). One-way analysis of variance (ANOVA) via Duncan’s test was used to compare the mean values. The differences were considered to be significant at *p* < 0.05. 

## 4. Conclusions

Despite the high variability between the tested cultivars, in terms of both the quality and the content of bioactive compounds, it should be stated that the consumption of European pear fruit could be beneficial to the health of the consumer due to their health-promoting properties. The extracts of pears described in the present study exhibited strong antioxidant and anti-inflammatory activity. Additionally, in all varieties, high antiproliferative activity was established against bladder cancer. Therefore, due to the high content of bioactive compounds, their strong health-promoting activity, and low cytotoxicity, the extracts may be used as a potential new source of bioactive polyphenols with possible applications in the production of functional food. In addition, knowledge of the chemical composition of the fruit used for processing will allow for selecting appropriate process parameters and avoid quality defects of the finished product.

## Figures and Tables

**Table 1:** Characteristics of fruits at harvest.

Features	Hortensia	Conference	Alexander Lucas	Nojabrska	Radana	Minimum	Maximum	Mean
Fresh weight (g)	183.92 ^a1^	213.59 ^b^	216.41 ^b^	324.15 ^c^	165.21 ^a^	165.21	324.15	220.75
Total soluble solids (°Brix)	9.35 ^a^	12.64 ^c^	11.10 ^b^	12.43 ^c^	10.69 ^b^	9.35	12.64	11.22
Titratable acidity (%)	0.39 ^d^	0.15 ^a^	0.31 ^c^	0.24 ^b^	0.47 ^e^	0.15	0.47	0.31
Blush (%)	30.33 ^b^	0.00 ^a^	0.00 ^a^	0.00 ^a^	46.67 ^c^	0.00	46.67	15.61
Firmness (N)	46.36 ^b^	50.42 ^c^	42.80 ^a^	59.57 ^d^	73.02 ^e^	42.80	73.02	54.49
Ethylene (µL∙kg^−1^∙h^−1^)	0.28 ^a^	9.98 ^b^	0.08 ^a^	0.04 ^a^	0.16 ^a^	0.04	9.98	3.50
CO_2_ (µL∙g^−1^∙h^−1^)	3.41 ^a^	10.63 ^d^	5.40 ^b^	4.84 ^b^	7.94 ^c^	3.41	10.63	7.64
Starch index (1–10)	9.80 ^d^	10.00 ^d^	8.73 ^c^	7.80 ^b^	5.53 ^a^	5.53	10.00	8.36

^1^ Data are expressed as mean (*n* = 9). Means followed by the same letter in the rows are not significantly different at *p* = 0.05 according to Duncan’s test.

## Supplementary Materials

The following are available online. Figure S1: Correlation coefficient calculated for the polyphenolic compounds and the PPO activity. Figure S2: Correlation coefficient calculated for the polyphenolic compounds and the DPPH activity. Figure S3: Correlation coefficient calculated for the polyphenolic compounds and the FRAP activity. Figure S4: Correlation coefficient calculated for the carotenoids and the DPPH antioxidant activity. Figure S5: Correlation coefficient calculated for the carotenoids and the FRAP antioxidant activity. Figure S6: Correlation coefficient calculated for the chlorophylls and the DPPH antioxidant activity. Figure S7: Correlation coefficient calculated for the chlorophylls and the FRAP antioxidant activity. Figure S8: Correlation coefficient calculated for the triterpenoids and the antioxidant activity (DPPH and FRAP). Table S1: Content of the polyphenolic compounds and the polyphenol oxidase activity in pear cultivars. Table S2: Content of the triterpenoids, chlorophylls, and carotenoids in pear cultivars. Table S3: Antioxidant activity of pear cultivars. Table S4: Anti-inflammatory properties of pear cultivars. Table S5: Antiproliferative properties of pear cultivars.
