# Supplementary material for: Bioactive Compounds and Health-Promoting Properties of Pear (Pyrus communis L.) Fruits
Source: Molecules. 2020 Sep 27;25(19):4444. doi: 10.3390/molecules25194444 (PMC7582546; doi:10.3390/molecules25194444)
Supplement: Supplementary file 1 [file molecules-25-04444-s001.pdf]

Supplementary Materials

Bioactive Compounds and Health-Promoting Properties of Pear (*Pyrus communis* L.) Fruits

Joanna Kolniak-Ostek [1,*], Dagmara Kłopotowska [2], Krzysztof P. Rutkowski [3], Anna Skorupińska [3] and Dorota E. Kruczyńska [3]

1 Department of Fruit, Vegetable and Plant Nutraceutical Technology, Wroclaw University of Environmental and Life Sciences, Chelmonskiego 37 Street, 51-630 Wroclaw, Poland
2 Laboratory of Experimental Anticancer Therapy, Institute of Immunology and Experimental Therapy, Polish Academy of Sciences, Weigla 12 Street, 53-114 Wroclaw, Poland; dagmara.klopotowska@hirszfeld.pl
3 Research Institute of Horticulture, Konstytucji 3 Maja 1/3 Street, 96-100 Skierniewice, Poland; krzysztof.rutkowski@inhort.pl (K.P.R.); anna.skorupinska@inhort.pl (A.S.); dorota.kruczynska@inhort.pl (D.E.K.)
* Correspondence: joanna.kolniak-ostek@upwr.edu.pl

Table S1. Content of polyphenolic compounds [mg/kg DW] and polyphenol oxidase activity [ΔU/g min] in pear cultivars.

	Hortensia	Conference	Alexander Lucas	Nojabrska	Radana	*minimum*	*maximum*	*mean*
Phenolic acids								
Caffeic acid and derivatives	936.92 ± 32.5 b[1]	550.42 ± 13.28 a	1167.68 ± 33.28 c	3423.89 ± 45.81 e	2183.61 ± 15.66 d	550.42	3423.89	1652.50
p-Coumaric acid and derivatives	57.82 ± 2.35 c	15.69 ± 0.55 b	2.45 ± 0.01 a	84.56 ± 3.99 d	56.80 ± 4.21 c	2.45	84.56	43.47
Ferulic acid derivatives	33.26 ± 1.26 b	0.00 ± 0.0 a	0.00 ± 0.0 a	0.00 ± 0.0 a	0.00 ± 0.0 a	0.00	33.26	6.65
Quinic acid	8.38 ± 0.31 b	0.00 ± 0.0 a	0.00 ± 0.0 a	0.00 ± 0.0 a	37.29 ± 1.12 c	0.00	37.29	9.13
Sinapic acid derivatives	201.58 ± 10.02 b	0.00 ± 0.0 a	0.00 ± 0.0 a	0.00 ± 0.0 a	0.00 ± 0.0 a	0.00	201.58	40.32
Syringic acid derivatives	582.82 ± 15.55 d	204.55 ± 8.27 c	87.09 ± 2.99 b	0.00 ± 0.0 a	2243.45 ± 18.17 e	0.00	2243.45	623.58
Sum	1820.78 ± 39.34 c	770.67 ± 21.00 a	1257.22 ± 30.2 b	3508.45 ± 41.82 d	4521.15 ± 36.92 e	770.67	4521.15	2375.65
Flavan-3-ols and procyanidines								
Monomeric catechins	871.26 ± 24.15d	593.38 ± 14.28 b	575.80 ± 15.87 b	664.05 ± 15.15 c	253.05 ± 8.99 a	253.05	871.26	591.51
Polymeric procyanidines	89.65 ± 3.55 a	142.95 ± 4.15 b	226.68 ± 7.54 c	444.69 ± 20.78 d	205.71 ± 8.02 c	89.65	444.69	221.93
Sum	960.90 ± 27.7 d	736.33 ± 18.43 b	802.48 ± 23.41 c	1108.74 ± 5.63 e	458.75 ± 17.01 a	458.75	1108.74	813.44
Flavonols								
Kaempferol derivatives	20.80 ± 1.25 b	17.00 ± 0.21 b	0.00 ± 0.0 a	3.86 ± 0.12 b	196.87 ± 6.69 c	0.00	196.87	47.70
Isorhamnetin derivatives	443.01 ± 10.94 d	328.22 ± 6.62 c	182.63 ± 9.15 b	102.66 ± 2.15 a	486.75 ± 17.89 e	102.66	486.75	308.65
Quercetin derivatives	407.81 ± 14.43 e	67.45 ± 2.78 b	122.26 ± 6.03 c	29.28 ± 4.12 a	372.59 ± 13.13 d	29.28	407.81	199.88
Sum	871.61 ± 26.62 d	412.67 ± 4.05 c	304.89 ± 15.18 b	135.79 ± 6.39 a	1056.20 ± 1.93 e	135.79	1056.20	556.23
Flavones								
Apigenin derivatives	89.10 ± 15.77 b	68.67 ± 1.89 a	101.10 ± 7.74 c	160.70 ± 8.01 d	158.05 ± 9.61 d	68.67	160.70	115.52
Hydrochalcones								
Arbutin	286.70 ± 20.69 c	200.59 ± 5.94 a	229.82 ± 8.11 b	212.48 ± 5.55 a	493.56 ± 13.00 d	200.59	493.56	284.63
Sum of phenolic compounds	4029.09 ± 116.27 c	2188.93 ± 14.25 a	2874.93 ± 110.36b	4946.74 ± 115.64 d	6687.71 ± 18.45 e	2188.93	6687.71	4438.32
PPO activity	487.86 ± 15.30 b	458.94 ± 17.80 a	542.47 ± 21.00 c	592.92 ± 22.60 d	739.80 ± 25.80 e	458.94	739.80	654.40

[1] **Data are expressed as mean ± SD (*n* = 9). Means followed by the same letter in the rows are not significantly different at *p* = 0.05 according to Duncan's test.**

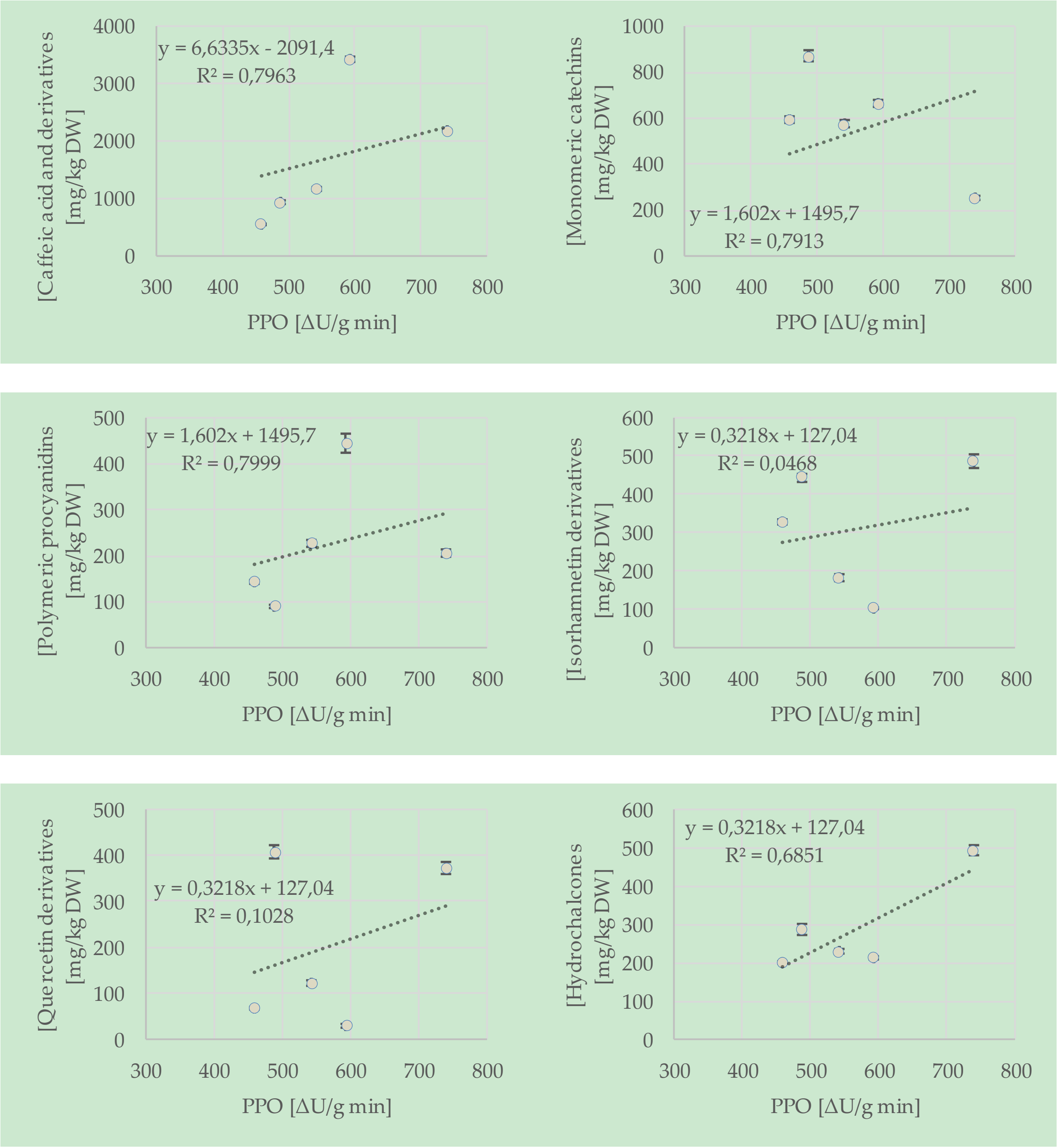

Figure S1. Correlation coefficient calculated for polyphenolic compounds and PPO activity. Data are expressed as mean ± SD (*n* = 9).

Table S2. Content of triterpenoids, chlorophylls and carotenoids [mg/kg DW] in pear cultivars.

	Hortensia	Conference	Alexander Lucas	Nojabrska	Radana	*minimum*	*maximum*	*mean*
Betulinic acid	52.90 ± 4.21 b[1]	77.63 ± 5.89 c	76.94 ± 6.32 c	116.12 ± 4.22 d	25.90 ± 1.12 a	25.90	116.12	69.90
Oleanolic acid	87.00 ± 4.89 c	41.16 ± 3.15 a	74.47 ± 5.88 b	239.34 ± 6.97 e	118.84 ± 5.83 d	41.16	239.34	112.16
Ursolic acid	370.71 ± 8.77 b	333.92 ± 9.01 a	451.95 ± 10.01 a	1190.71 ± 16.10 e	1107.73 ± 11.11 d	333.92	1190.71	691.00
Sum of triterpenoids	510.61 ± 8.09 b	452.71 ± 0.03 a	603.36 ± 2.19 c	1546.17 ± 27.29 e	1252.47 ± 15.82d	452.71	1546.17	873.06
Pheophorbide a	0.26 ± 0.00 b	0.18 ± 0.00 a	0.58 ± 0.00 c	0.18 ± 0.00a	0.67 ± 0.00 d	0.18	0.67	0.37
Chlorophyllide b	2.26 ± 0.10 b	0.00 ± 0.00 a	2.44 ± 0.10 b	7.45 ± 0.52 d	5.22 ± 0.09 c	0.00	7.45	3.47
Chlorophyllide a	76.73 ± 2.35 c	114.29 ± 5.25 e	56.76 ± 1.99 b	34.71 ± 1.89 a	107.93 ± 5.22 d	34.71	114.29	78.08
Chlorophyll b	0.67 ± 0.00 b	0.79 ± 0.00 c	0.43 ± 0.00 a	0.37 ± 0.00 a	0.81 ± 0.00 c	0.37	0.81	0.61
Chlorophyll a [§]	0.86 ± 0.00 c	0.92 ± 0.00 c	0.66 ± 0.00 b	0.37 ± 0.00 a	0.93 ± 0.00 c	0.37	0.93	0.75
Pheophitine b	79.72 ± 3.12 b	85.85 ± 6.21 c	15.31 ± 1.02 a	16.24 ± 0.45 a	124.65 ± 4.38 d	15.31	124.65	64.35
Pheophitine a	32.19 ± 1.05 c	34.34 ± 2.89 c	12.37 ± 0.87 a	18.10 ± 0.71 b	51.53 ± 1.11 d	12.37	51.53	29.70
Sum of chlorophylls	192.68 ± 1.76 c	236.37 ± 1.93 d	88.55 ± 3.98 b	77.43 ± 1.11 a	291.74 ± 1.86 e	77.43	291.74	177.35
all-*trans*-Lutein	4.40 ± 0.21 c	4.69 ± 0.15 c	1.69 ± 0.01 a	2.47 ± 0.01 b	7.04 ± 0.44 d	1.69	7.04	4.06
13-*cis*-Lutein	5.39 ± 0.66 d	4.80 ± 0,67 c	2.98 ± 0.01 a	3.28 ± 0.02 b	7.87 ± 0.29 e	2.98	7.87	4.87
all-*trans* β-Carotene	5.70 ± 0.36 b	1.67 ± 0.00 a	1.54 ± 0.00 a	12.58 ± 0.86 c	13.98 ± 1.47 d	1.54	13.98	7.09
9-*cis*-β-Carotene	63.99 ± 2.28 d	26.35 ± 1.01 ab	23.70 ± 1.71 a	52.54 ± 1.56 c	117.72 ± 3.30 e	23.70	117.72	56.86
Sum of carotenoids	79.48 ± 3.51 d	37.52 ± 0.19 b	29.91 ± 1.00 a	70.87 ± 2.45 c	146.61 ± 1.98 e	29.91	146.61	72.88

[1] Data are expressed as mean ± SD (n = 9). Means followed by the same letter in the rows are not significantly different at p = 0.05 according to Duncan's test.

Table S3. Antioxidant activity of pear cultivars [mMol Trolox/kg DW].

	**Hortensia**	**Conference**	**Alexander Lucas**	**Nojabrska**	**Radana**	***minimum***	***maximum***	***mean***
DPPH	4.96 ± 0.16 c[1]	3.98 ± 0.10 a	4.42 ± 0.21 b	5.42 ± 0.21 d	7.70 ± 0.26 e	3.98	7.70	4.30
FRAP	11.54 ± 0.31 d	4.37 ± 0.11 a	5.40 ± 0.12 b	9.40 ± 0.24 c	15.64 ± 0.33 e	4.37	15.64	9.27

[1] **Data are expressed as mean ± SD (n = 9). Means followed by the same letter in the rows are not significantly different at p = 0.05 according to Duncan's test.**

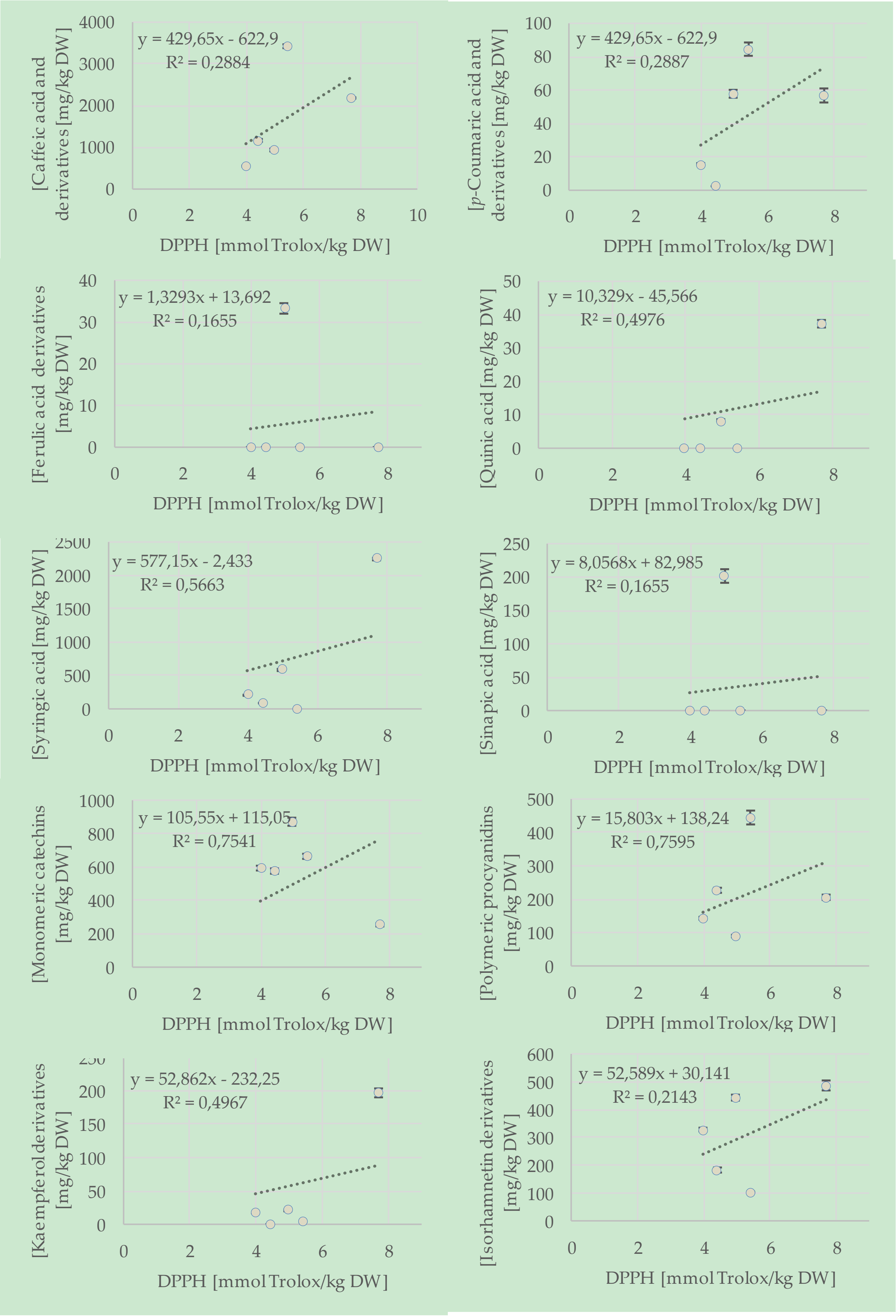

y = 429,65x - 622,9
R² = 0,2884
[Caffeic acid and derivatives [mg/kg DW]]
DPPH [mmol Trolox/kg DW]
y = 429,65x - 622,9
R² = 0,2887
[p-Coumaric acid and derivatives [mg/kg DW]]
DPPH [mmol Trolox/kg DW]
y = 1,3293x + 13,692
R² = 0,1655
[Ferulic acid derivatives [mg/kg DW]]
DPPH [mmol Trolox/kg DW]
y = 10,329x - 45,566
R² = 0,4976
[Quinic acid [mg/kg DW]]
DPPH [mmol Trolox/kg DW]
y = 577,15x - 2,433
R² = 0,5663
[Syringic acid [mg/kg DW]]
DPPH [mmol Trolox/kg DW]
y = 8,0568x + 82,985
R² = 0,1655
[Sinapic acid [mg/kg DW]]
DPPH [mmol Trolox/kg DW]
y = 105,55x + 115,05
R² = 0,7541
[Monomeric catechins [mg/kg DW]]
DPPH [mmol Trolox/kg DW]
y = 15,803x + 138,24
R² = 0,7595
[Polymeric procyanidins [mg/kg DW]]
DPPH [mmol Trolox/kg DW]
y = 52,862x - 232,25
R² = 0,4967
[Kaempferol derivatives [mg/kg DW]]
DPPH [mmol Trolox/kg DW]
y = 52,589x + 30,141
R² = 0,2143
[Isorhamnetin derivatives [mg/kg DW]]
DPPH [mmol Trolox/kg DW]

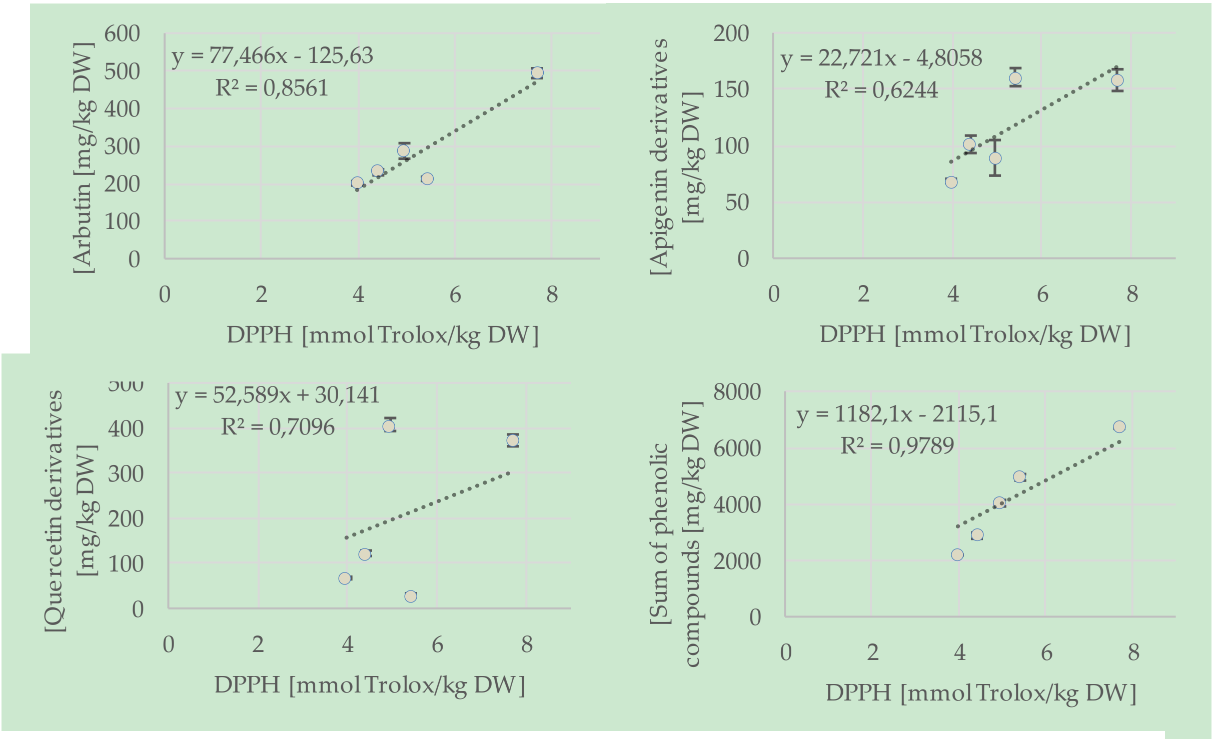

Figure S2. Correlation coefficient calculated for polyphenolic compounds and DPPH activity. Data are expressed as mean ± SD ($n = 9$).

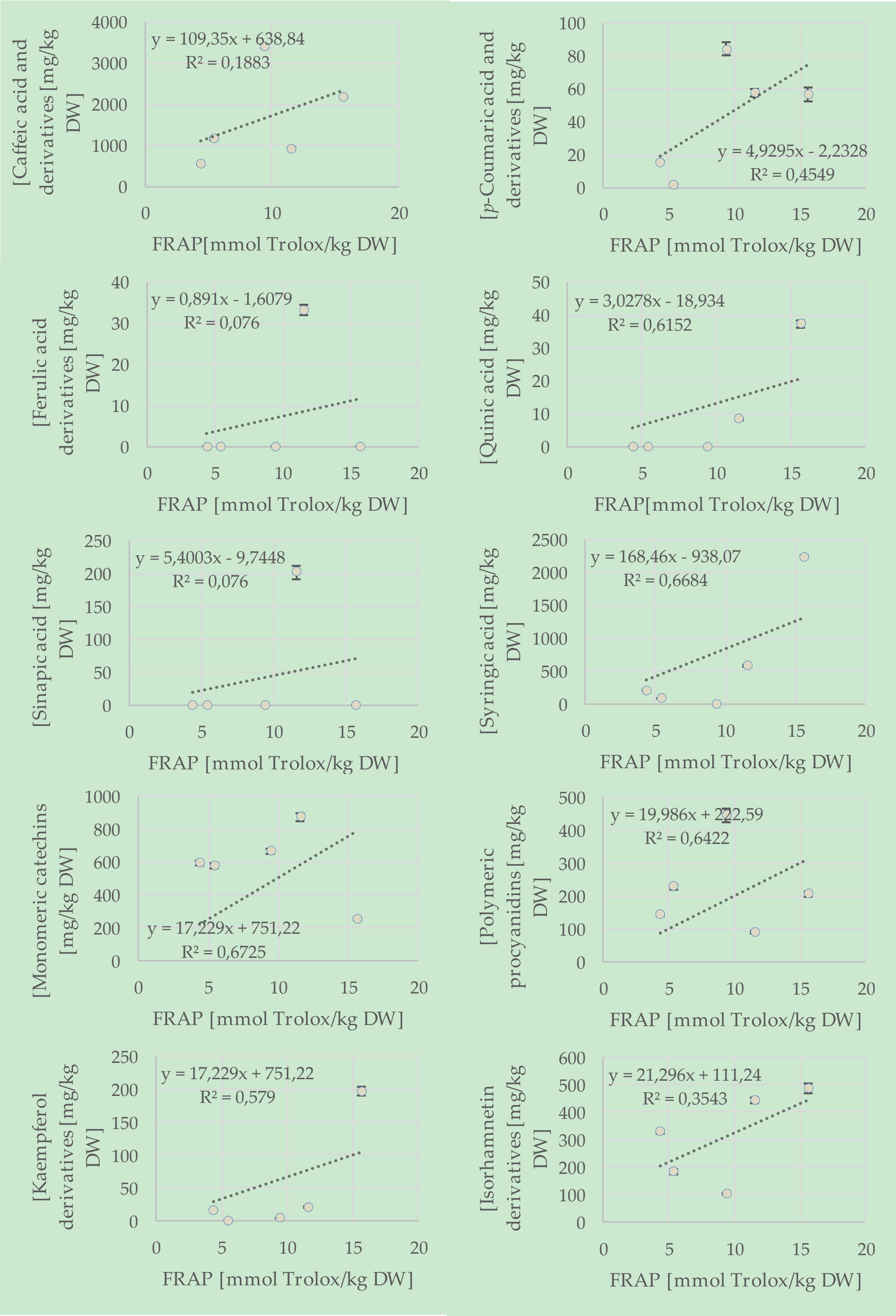
[Caffeic acid and derivatives [mg/kg DW]
y = 109,35x + 638,84
R² = 0,1883
FRAP[mmol Trolox/kg DW]
[p-Coumaric acid and derivatives [mg/kg DW]
y = 4,9295x - 2,2328
R² = 0,4549
FRAP [mmol Trolox/kg DW]
[Ferulic acid derivatives [mg/kg DW]
y = 0,891x - 1,6079
R² = 0,076
FRAP [mmol Trolox/kg DW]
[Quinic acid [mg/kg DW]
y = 3,0278x - 18,934
R² = 0,6152
FRAP[mmol Trolox/kg DW]
[Sinapic acid [mg/kg DW]
y = 5,4003x - 9,7448
R² = 0,076
FRAP [mmol Trolox/kg DW]
[Syringic acid [mg/kg DW]
y = 168,46x - 938,07
R² = 0,6684
FRAP [mmol Trolox/kg DW]
[Monomeric catechins [mg/kg DW]
y = 17,229x + 751,22
R² = 0,6725
FRAP [mmol Trolox/kg DW]
[Polymeric procyanidins [mg/kg DW]
y = 19,986x + 222,59
R² = 0,6422
FRAP [mmol Trolox/kg DW]
[Kaempferol derivatives [mg/kg DW]
y = 17,229x + 751,22
R² = 0,579
FRAP [mmol Trolox/kg DW]
[Isorhamnetin derivatives [mg/kg DW]
y = 21,296x + 111,24
R² = 0,3543
FRAP [mmol Trolox/kg DW]

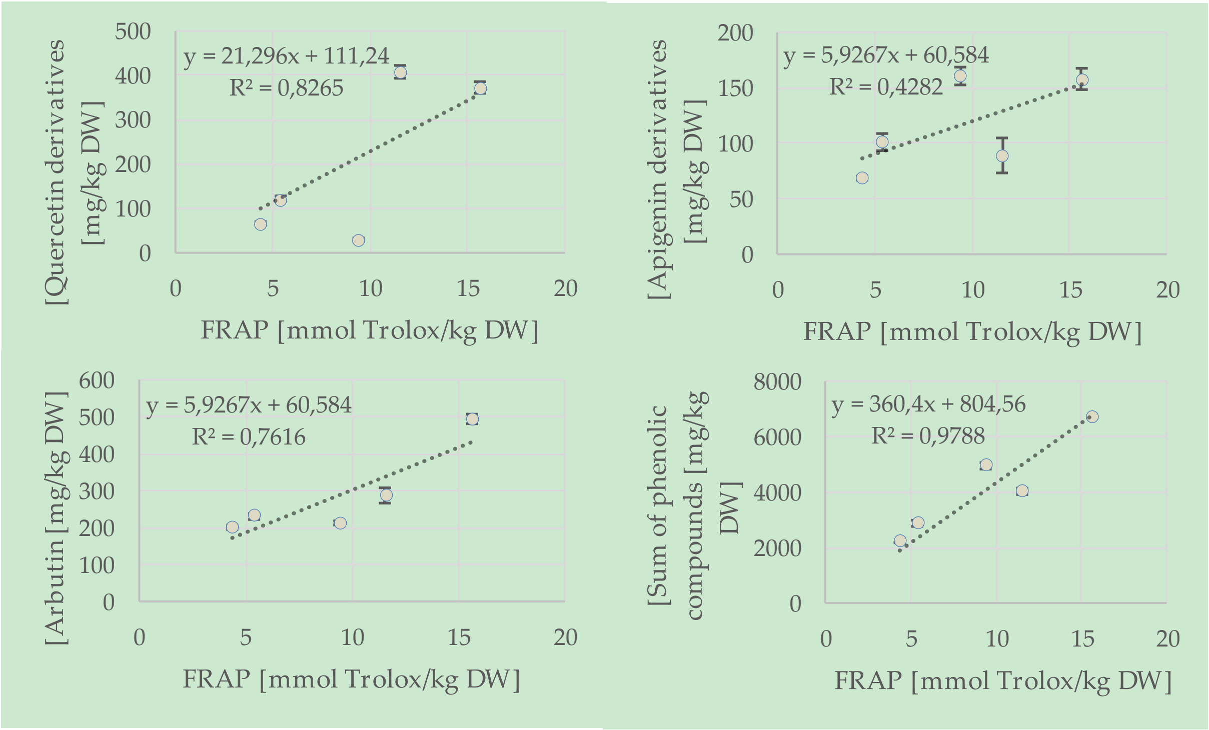

Figure S3. Correlation coefficient calculated for polyphenolic compounds and FRAP activity. Data are expressed as mean ± SD (*n* = 9).

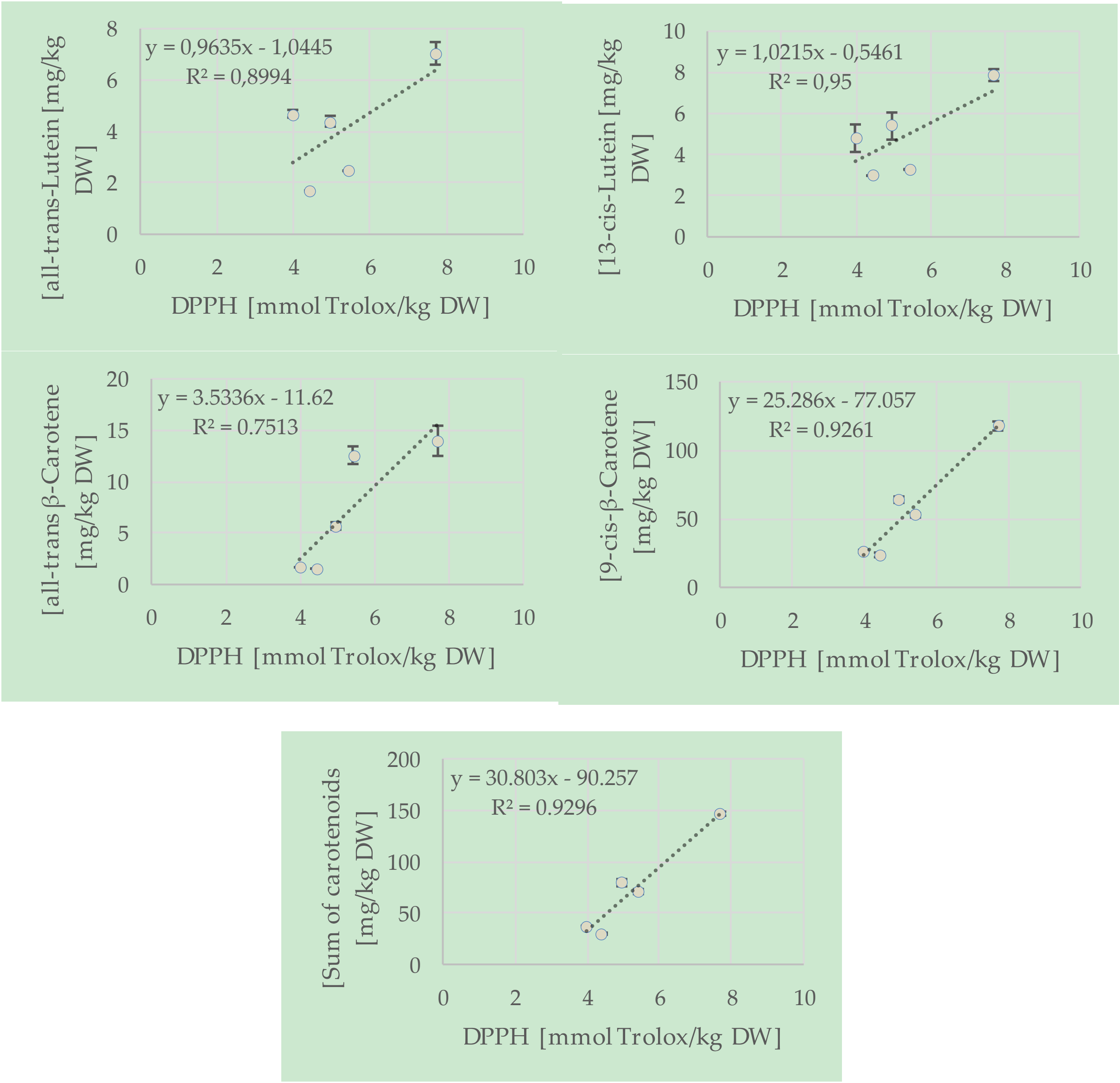

Figure S4. Correlation coefficient calculated for carotenoids and DPPH antioxidant activity. Data are expressed as mean ± SD (n = 9).

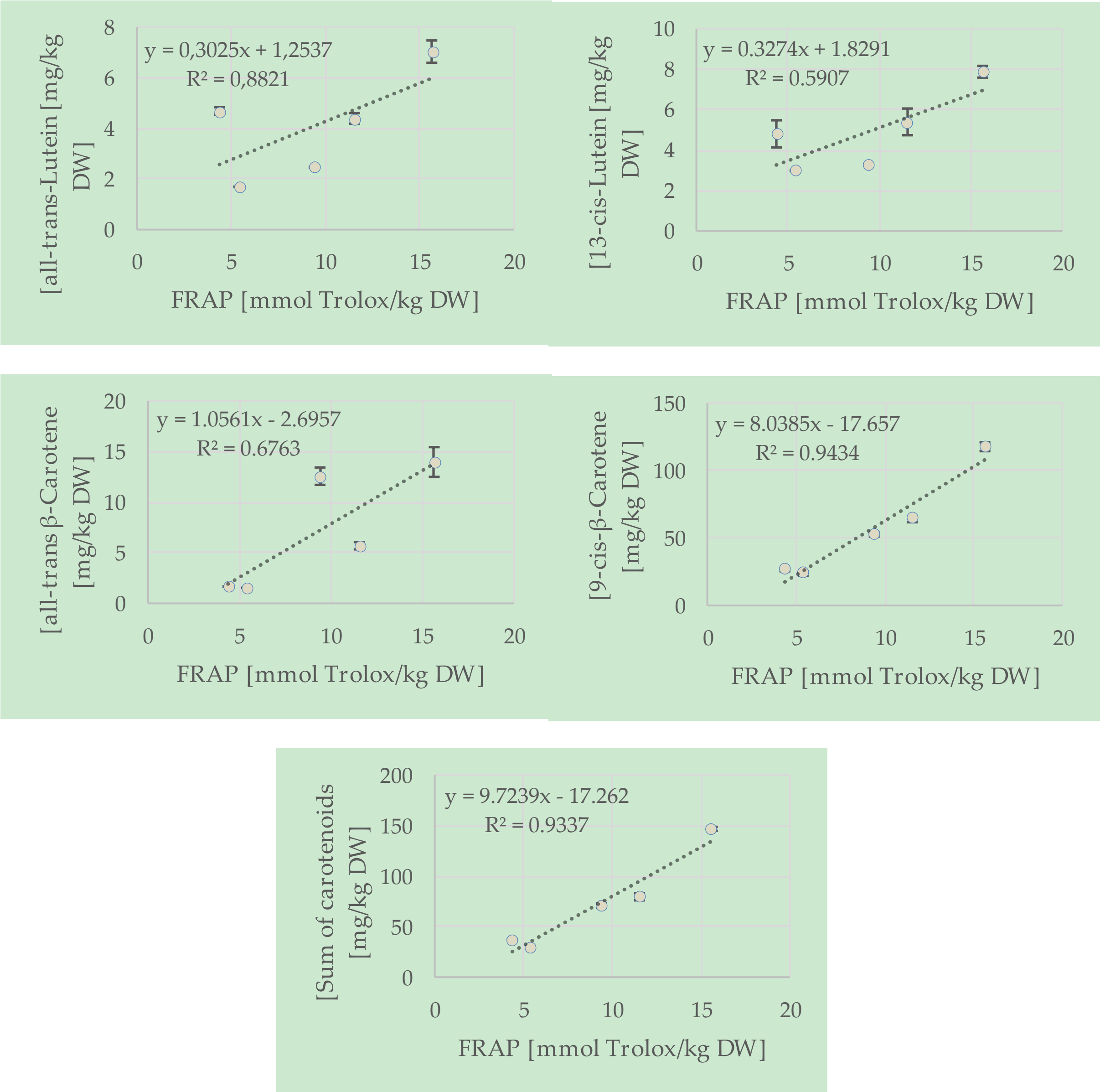

Figure S5. Correlation coefficient calculated for carotenoids and FRAP antioxidant activity. Data are expressed as mean ± SD (*n* = 9).

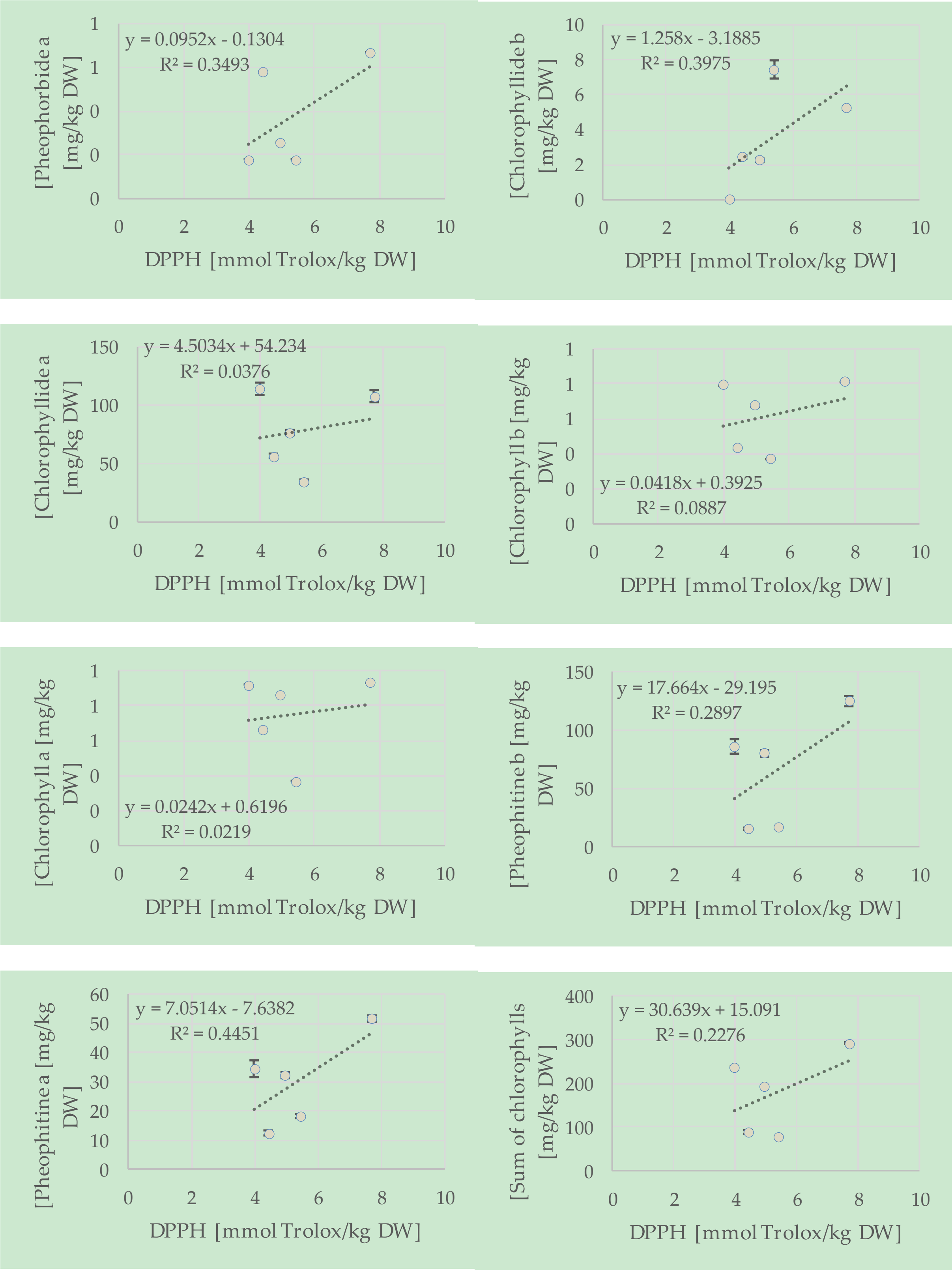

Figure S6. Correlation coefficient calculated for chlorophylls and DPPH antioxidant activity. Data are expressed as mean ± SD (n = 9).

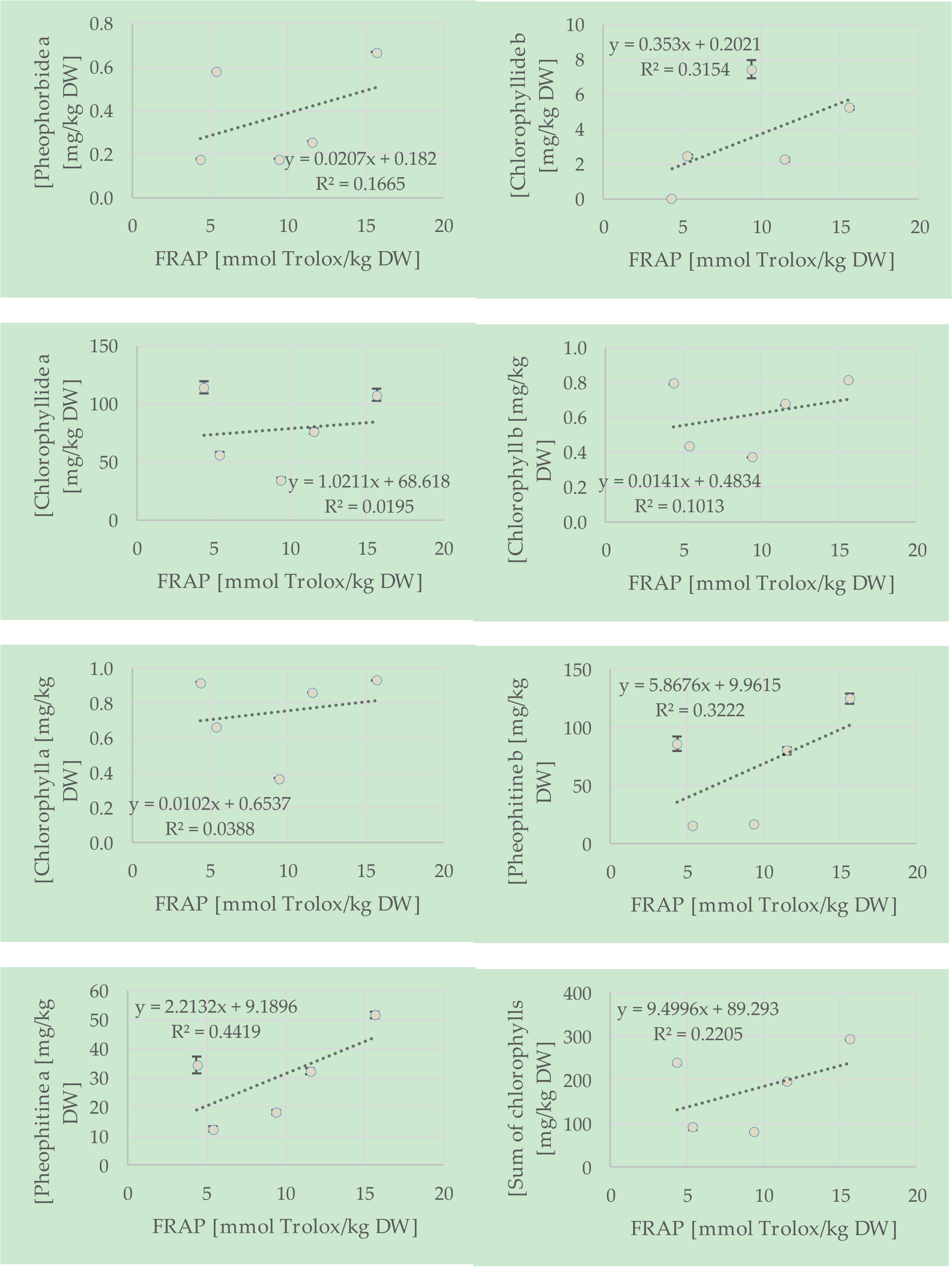

Figure S7. Correlation coefficient calculated for chlorophylls and FRAP antioxidant activity. Data are expressed as mean ± SD (n = 9).

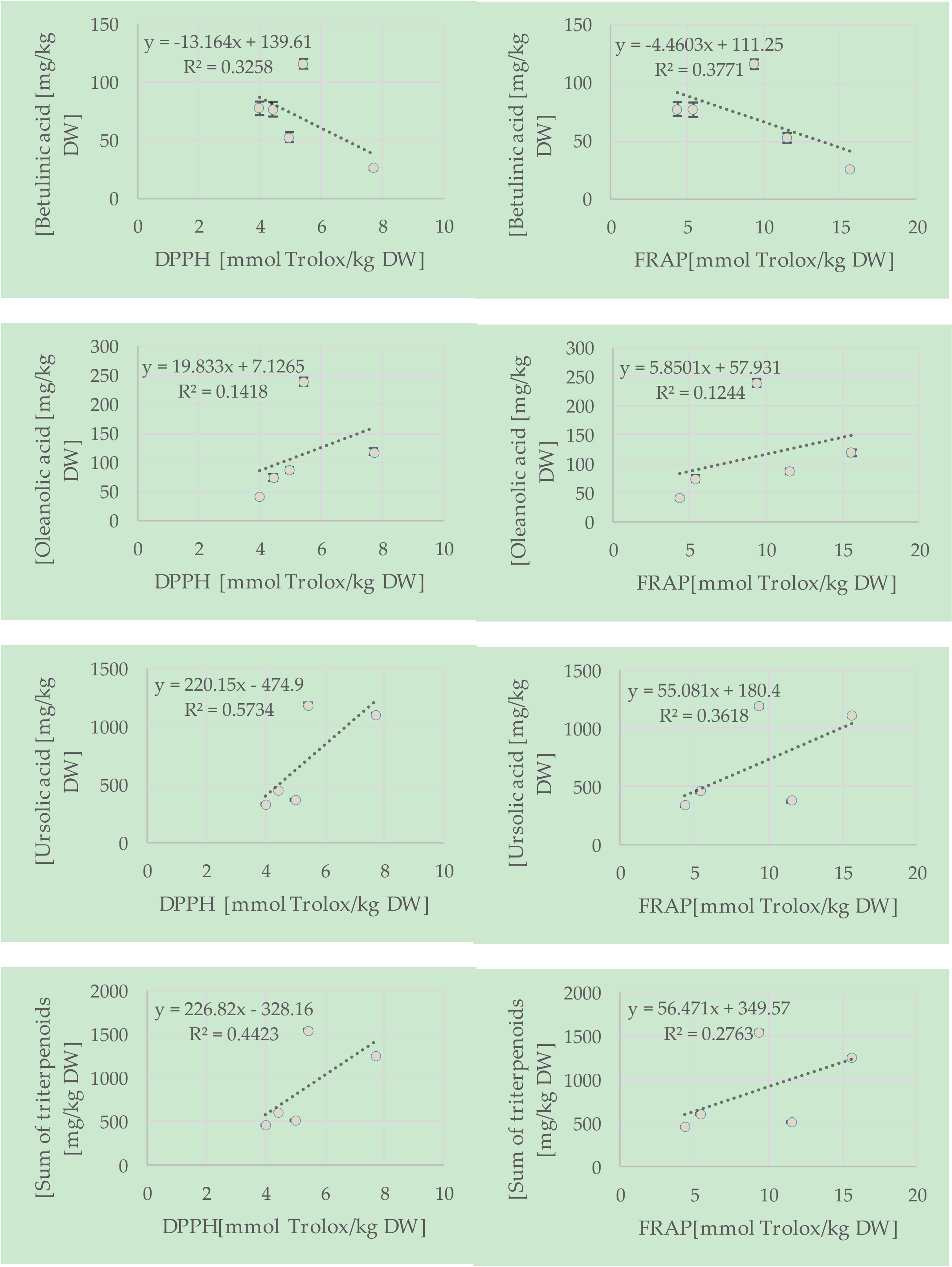

Figure S8. Correlation coefficient calculated for triterpenoids and antioxidant activity (DPPH and FRAP). Data are expressed as mean ± SD (n = 9).

Table S4. Anti-inflammatory properties of pear cultivars [% of inhibition].

	**Hortensia**	**Conference**	**Alexander Lucas**	**Nojabrska**	**Radana**	***minimum***	***maximum***	***mean***
COX-1	38.91 ± 0.52 a[1]	74.02 ± 1.23 b	33.83 ± 0.67 a	31.95 ± 0.33 a	75.93 ± 1.00 b	31.95	75.93	50.93
COX-2	45.43 ± 0.68 b	81.88 ± 1.11 c	38.52 ± 1.51 a	34.14 ± 1.27 a	90.80 ± 1.32 d	34.14	90.80	58.15

[1] **Data are expressed as mean ± SD (*n* = 9). Means followed by the same letter in the rows are not significantly different at *p* = 0.05 according to Duncan's test.**

Table S5. Antiproliferative properties of pear cultivars [IC50].

	**Hortensia**	**Conference**	**Alexander Lucas**	**Nojabrska**	**Radana**	***maximum***	***minimum***	***mean***
A498	2.85 ± 0.57c[1]	3.19 ± 0.13 d	3.31 ± 0.02 d	3.30 ± 0.11 d	1.81 ± 0.76 a	*3.31*	*1.81*	*2.89*
A549	2.22 ± 0.69 d	0.43 ± 0.21 a	2.52 ± 0.25 d	1.86 ± 0.70 c	1.02 ± 0.41 b	*2.52*	*0.43*	*1.61*
HCV29T	0.68 ± 0.23 c	0.32 ± 0.00 a	1.43 ± 0.47 e	1.06 ± 0.25 d	0.64 ± 0.10 c	*1.43*	*0.32*	*0.83*
HT-29	2.42 ± 0.19 e	2.11 ± 0.75 d	1.61 ± 0.32 c	1.14 ± 0.18 b	0.44 ± 0.08 a	*2.42*	*0.44*	*1.54*
LNCaP	1.48 ± 0.31 e	0.97 ± 0.83 b	1.37 ± 0.51 d	1.16 ± 0.20 c	0.63 ± 0.19 a	*1.48*	*0.63*	*1.12*
MCF-7	1.86 ± 0.67 b	0.39 ± 0.08 a	2.23 ± 0.41 c	2.18 ± 0.55 c	2.14 ± 0.54 c	*2.23*	*0.39*	*1.76*

[1] **Data are expressed as mean ± SD (n = 9). Means followed by the same letter in the rows are not significantly different at p = 0.05 according to Duncan's test.**
